# Unveiling dysregulated lncRNAs and networks in non-syndromic cleft lip with or without cleft palate pathogenesis

**DOI:** 10.1038/s41598-024-51747-8

**Published:** 2024-01-10

**Authors:** Caihong Wu, Haojie Liu, Zhuorong Zhan, Xinyu Zhang, Mengnan Zhang, Jiawen You, Junqing Ma

**Affiliations:** 1 Jiangsu Key Laboratory of Oral Diseases, Nanjing Medical University, Nanjing, China; 2 Department of Orthodontics, Affiliated Hospital of Stomatology, Nanjing Medical University, Nanjing, China; 3 Stomatological Hospital affiliated Suzhou Vocational Health College, Suzhou, China

**Keywords:** Computational biology and bioinformatics, Developmental biology, Genetics

## Abstract

Non-syndromic cleft lip with or without cleft palate (NSCL/P) is a common congenital facial malformation with a complex, incompletely understood origin. Long noncoding RNAs (lncRNAs) have emerged as pivotal regulators of gene expression, potentially shedding light on NSCL/P's etiology. This study aimed to identify critical lncRNAs and construct regulatory networks to unveil NSCL/P's underlying molecular mechanisms. Integrating gene expression profiles from the Gene Expression Omnibus (GEO) database, we pinpointed 30 dysregulated NSCL/P-associated lncRNAs. Subsequent analyses enabled the creation of competing endogenous RNA (ceRNA) networks, lncRNA-RNA binding protein (RBP) interaction networks, and lncRNA cis and trans regulation networks. RT-qPCR was used to examine the regulatory networks of lncRNA in vivo and in vitro. Furthermore, protein levels of lncRNA target genes were validated in human NSCL/P tissue samples and murine palatal shelves. Consequently, two lncRNAs and three mRNAs: *FENDRR* (log2FC = − 0.671, P = 0.040), *TPT1-AS1* (log2FC = 0.854, P = 0.003), *EIF3H* (log2FC = − 1.081, P = 0.041), *RBBP6* (log2FC = 0.914, P = 0.037), and *SRSF1* (log2FC = 0.763, P = 0.026) emerged as potential contributors to NSCL/P pathogenesis. Functional enrichment analyses illuminated the biological functions and pathways associated with these lncRNA-related networks in NSCL/P. In summary, this study comprehensively delineates the dysregulated transcriptional landscape, identifies associated lncRNAs, and reveals pivotal sub-networks relevant to NSCL/P development, aiding our understanding of its molecular progression and setting the stage for further exploration of lncRNA and mRNA regulation in NSCL/P.

## Introduction

Non-syndromic cleft lip with or without cleft palate (NSCL/P) is a relatively common congenital malformation of the facial structure, characterized by a complex and incompletely understood pathogenesis^1^. Notably, NSCL/P exhibits particularly high incidence rates in Asian populations compared to other ethnic groups. Generally, Asian and Amerindian populations report the highest birth prevalence rates, often reaching 1/500, while European-derived populations present intermediate prevalence rates around 1/1000. In contrast, African-derived populations exhibit the lowest prevalence rates, approximately 1/2500. These variations in prevalence suggest that the relative contribution of individual susceptibility genes may differ among various populations^2^. Therefore, it is imperative to explore the etiology of NSCL/P in Asian populations, characterized by high incidence rates, to gain a more comprehensive understanding of the genetic and environmental factors influencing this complex condition.

The multifactorial etiology of NSCL/P involves both genetic and environmental factors, with moderate recurrence rates that impose substantial financial burdens on affected families and society^1,3^. Notably, processes such as cell proliferation, differentiation, adhesion, migration, apoptosis and epithelial-mesenchymal transition (EMT) are pivotal in palatogenesis, and their dysregulation may contribute to NSCL/P^4^. Multiple candidate genes, including *IRF6*^5^, *FOXE1*, *GLI2*, *JAG2*, *LHX8*, *MSX1*, *MSX2*, *SATB2*, *SKI*, *SPRY2*, *TBX10*^6^ and *SH3PXD2A*^7^, have been identified in relation to the deregulation of these processes in NSCL/P. Furthermore, emerging evidence suggests that certain long noncoding RNAs (lncRNAs), such as *H19*^8^, *MEG3*^9^, *MALAT1*, *NEAT1*^10^, *ZFAS1*^11^, and *RP11-462G12. 2*^12^, exhibit aberrant expression in NSCL/P. These lncRNAs, exceeding 200 nucleotides in length, emanate from non-coding regions within various genes^13^. Contrary to the past notion of non-coding RNAs as mere transcriptional noise or non-functional sequences, contemporary research has unveiled their substantial roles in a plethora of biological processes^14^. LncRNAs, in particular, have attracted considerable attention due to their prevalence and multifaceted functions. In the non-coding transcriptome and transcribed sequences of mammals, lncRNAs are emerging as significant components. Despite several publications focusing on lncRNAs and their associated networks in the NSCL/P context, existing studies have predominantly emphasized the role of lncRNAs as miRNA sponges. This perspective may have inadvertently obscured other vital functions that these lncRNAs could perform within the NSCL/P context. A noteworthy observation is that while lncRNAs have been implicated in NSCL/P, the functional roles of these lncRNAs and the intricate regulatory mechanisms governing their participation in NSCL/P development remain largely unvalidated. These limitations underscore the compelling need for a more comprehensive comprehension of the lncRNA landscape and the specific regulatory networks within the realm of NSCL/P.

The function of long noncoding RNAs (lncRNAs) is closely tied to their subcellular localization. In the cytoplasm, lncRNAs exhibit regulatory roles by functioning as miRNA sponges, engaging in the competing endogenous RNA (ceRNA) mechanism^15^. Furthermore, cytoplasmic lncRNAs can interact with RNA binding proteins (RBPs), thereby exerting their biological effects^16^. On the other hand, nuclear localization of lncRNAs enables them to modulate gene transcription or pre-transcriptional processes through interactions with DNA promoter regions or transcription factors^17^, referred to as cis- and trans-regulation mechanisms^18^. Elucidating the precise subcellular localization of lncRNAs provides crucial insights into their functional roles and regulatory mechanisms.

In this study, we identified the aberrantly expressed lncRNAs of NSCL/P patients.

Subsequently, we synthetically analyzed multiple databases to predict the subcellular localization of lncRNAs and establish several lncRNA-centered regulatory networks in NSCL/P. Moreover, we used two cell lines, human embryonic palatal mesenchyme (HEPM) cells and human oral keratinocyte (HOK) cells to preliminarily verify the screened lncRNA regulatory network. In vivo, we employed NSCL/P tissues and murine palatal shelves to test the expression of differentially expressed genes.

## Materials and methods

### Flow chart for this study

**Figure 1:**
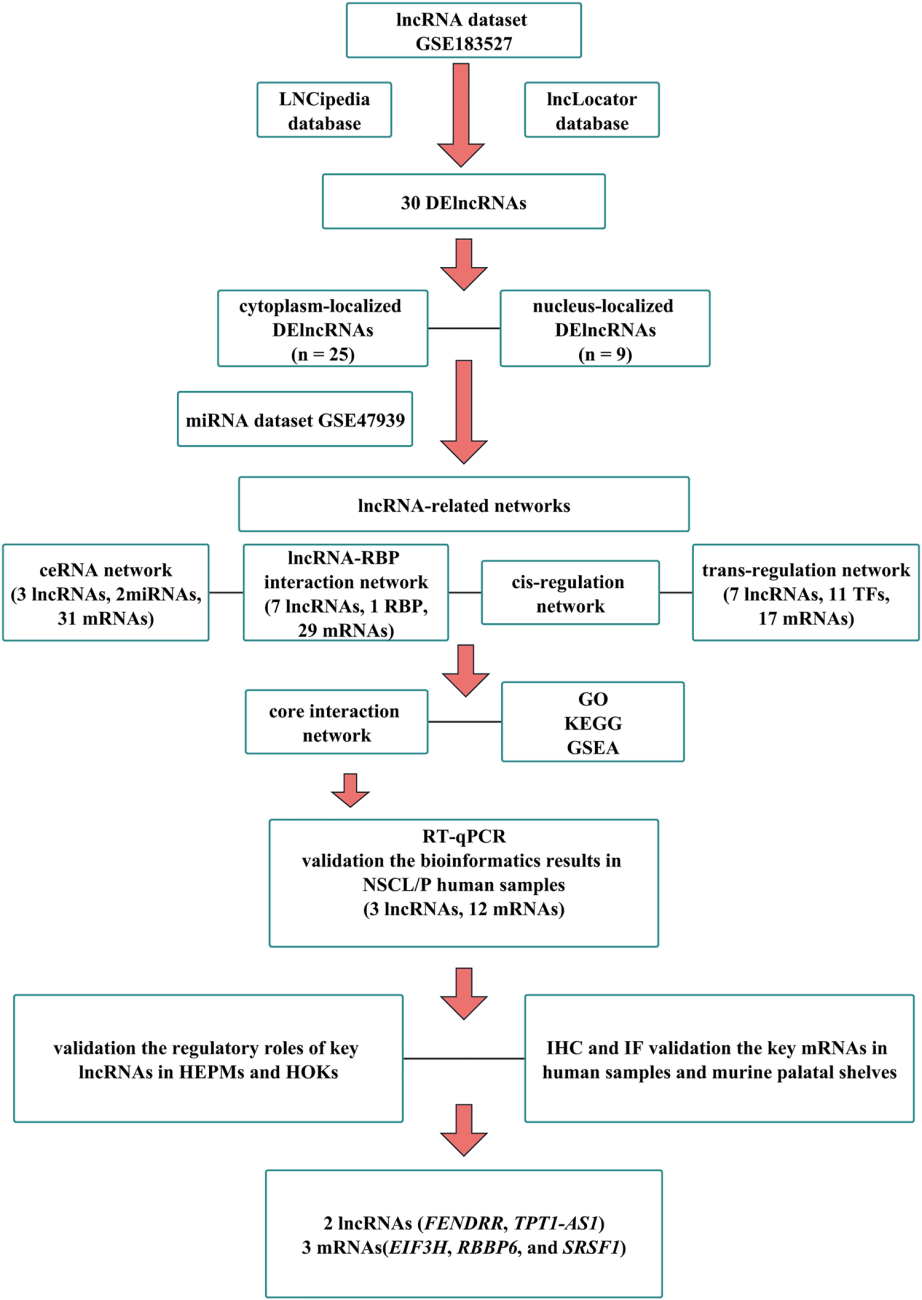
Flow chart for this study.

Figure 1 depicts the comprehensive research framework undertaken in this study. Initially, microarray dataset GSE183527 was obtained from the GEO database to identify differentially expressed lncRNAs (DElncRNAs) in non-syndromic cleft lip with or without cleft palate (NSCL/P). Subsequently, LNCipedia was utilized for annotation, and the lncLocator database aided in predicting the subcellular localization of the DElncRNAs. Thirty DElncRNAs were selected, with 21 localized in the cytoplasm, 5 in the nucleus, and 4 in both cellular compartments. In the subsequent phase, microarray dataset GSE47939 and a multi-database approach were employed to construct a ceRNA network, a lncRNA-RBP interaction network, and lncRNA cis and trans regulation networks. To delve into the pivotal role of DElncRNAs and their target DEmRNAs, a core interaction network was established. Functional enrichment analysis and gene set enrichment analysis of the target DEmRNAs were performed to glean insights into their biological significance. Finally, experimental validation was carried out using RT-qPCR on human NSCL/P tissue samples. Two cell lines, human embryonic palatal mesenchyme (HEPM) cells and human oral keratinocyte (HOK) cells, were employed to verify the screened lncRNA regulatory network in vitro. In vivo validation utilized NSCL/P tissues and murine palatal shelves to assess the protein-level expression of differentially expressed genes. Two lncRNAs (*FENDRR* and *TPT1-AS1*) and three mRNAs (*EIF3H*, *RBBP6*, and *SRSF1*) were successfully validated, suggesting their potential association with NSCL/P development.

### Microarray dataset collection and data process

The lncRNA/miRNA/mRNA expression profiles were obtained from the GEO database (https://www.ncbi.nlm.nih.gov/geo/)^19^. The lncRNA/mRNA dataset GSE183527 is based on GPL26963 (Agilent-085982 Arraystar human lncRNA V5 microarray). This dataset contains 6 tissue samples, including trimmed wound edge tissues and adjacent normal tissues of 3 NSCL/P human samples. The miRNA dataset GSE47939 is from GPL11487 (Agilent-021827 Human miRNA Microarray) and included 10 palate tissues from non-syndromic cleft palate patients and six from healthy controls. Details of each dataset, including the sample descriptions, are provided in Table S1. We utilized the “GEOquery”^20^ package of the R software (version 4. 2. 1, http://rproject.org/) to download the sample source from the GEO database. The raw data of GSE183527 and GSE47939 were downloaded, and we used the R package “limma”^21^ to perform background correction and quantile normalization. The R package “AnnoProbe” was employed to annotate gene symbols for probes in the two datasets. Following clearance, standardization, and annotation, the expression profile data from datasets GSE183527 and GSE47939 were utilized for bioinformatic analysis. Figures S1 and S2 show the raw data were normalized.

### NSCL/P-related differentially expressed (DE) RNAs screening

To determine the DERNAs between NSCL/P and healthy control, we applied the “BH”^22^ method of the “limma” R package with the following cutoff for adjustment: |log2FC|> 0.585, P-value < 0.05, and the FDR < 1 for GSE183527; |log2FC|> 0.585, P-value < 0.05, and the FDR < 0.3 for GSE47939. The volcano map and heat map were applied to visualize the DERNAs using the “ggplot2”^23^ and “pheatmap”^24^ R packages.

### Prediction of the subcellular localization and annotation of lncRNAs

The LNCipedia database (https://lncipedia.org/)^25^ was used to annotate lncRNAs, including their different transcript IDs and corresponding sequences, chromosomal locations, and classifications. Followed by obtaining the sequences of the significant lncRNAs, lncLocator (http://www.csbio.sjtu.edu.cn/bioinf/lncLocator/)^26^ was utilized to predict the subcellular localizations of these lncRNAs. The LncLocator algorithm generated a prediction score for each potential subcellular localization of lncRNA, including cytoplasm, nucleus, ribosome, cytosol, and exosome, and the highest score was taken as the predicted location.

### Construction of the DElncRNAs-related networks

According to the ceRNA hypothesis^15^, we constructed the ceRNA networks of the cytoplasm-localized DElncRNAs. First, the ENCORI database^27^ was used to predict the lncRNA-miRNA interaction pairs. Subsequently, followed by filtering out the lncRNA-miRNA interaction pairs, the R package “multiMiR”^28^ was applied to predict the target genes of the obtained miRNAs.

LncRNAs are associated with a plethora of cellular functions, most of which require interaction with one or more RNA-binding proteins (RBPs)^16^. RBPs are proteins known for their capability to bind to specific RNAs, playing a pivotal role in regulating gene expression at the RNA level^29^. It has been documented that lncRNAs can specifically bind to RBPs, influencing the functions of the RBPs^30^. Conversely, certain RBPs can interact with lncRNAs, modulating their functions and thereby regulating downstream gene expression^31^. Furthermore, RBPs not only impact the stability of lncRNAs but also influence their transport and localization^32^. This prompted us to profile the cytoplasm-localized DElncRNAs-RBP interaction network. We utilized the ENCORI database to predict the RBPs that interacted with the DElncRNAs. The co-expression target genes were filtered using Spearman’s correlation analysis, with a standard of |r_s_|> 0.9 and P-value < 0.05, between the DElncRNAs and DEmRNAs. Spearman’s rank correlation coefficient, a nonparametric rank statistic, was employed as a measure of the strength of the association between the two variables. Unlike Pearson correlation, Spearman’s rank correlation is distribution-free and suitable for both linear and nonlinear relationships. It evaluates the extent to which an arbitrary monotonic function can describe the relationship between two variables without assuming any specific frequency distribution. The coefficient ranges from − 1 to 1, where 1 indicates a perfect positive monotonic relationship, − 1 indicates a perfect negative monotonic relationship, and 0 indicates no monotonic relationship^33–35^. The application of Spearman's correlation allowed us to examine the presence of a consistent monotonic relationship between the expression levels of a specific lncRNA and a particular mRNA across samples^36,37^. It is crucial to note that this correlation does not infer direct physical interactions but rather suggests potential regulatory relationships. These findings require additional experimental validation and functional studies to elucidate the underlying biological mechanisms.

When lncRNAs are localized in the nucleus, they are known to regulate gene transcription or pre-transcription levels by binding to the DNA promoter region or transcription factors. This can occur in two ways: cis-regulation or trans-regulation^18^. For cis-regulation prediction, we filtered out the nucleus-localized DElncRNAs-DEmRNAs co-expressed pairs with Spearman’s correlation coefficient > 0.8, P-value < 0.05, and closely related genomic loci within 100 kb. For trans-regulation prediction, we used a screening criterion of Spearman’s correlation coefficient > 0.9, P-value < 0.05^38^. Moreover, emerging research highlights the role of lncRNAs as molecular scaffolds, orchestrating the recruitment of transcription factors (TFs) to the promoter regions of target genes^39^. It is well-documented that many TFs exhibit dual functions, acting as activators or repressors depending on various factors such as sequence specificity, chromatin structure, and modulatory elements. Notably, lncRNAs can modulate the effects of TFs, either enhancing or attenuating their impact, and in some cases, even reversing their regulatory actions^18,40^. In our pursuit of potential interacting proteins of nucleus-localized DElncRNAs, we extensively utilized the ENCORI database. Our selection criteria focused on proteins that not only demonstrated interaction with nucleus-localized lncRNAs but also functioned as TFs for the DEmRNAs. These selected TFs were obtained from the GeneCards database (https://www.genecards.org/)^41^. Finally, the DElncRNA-associated networks were visualized using Cytoscape version 3.9.1 software^42^.

### Functional enrichment analysis and gene set enrichment analysis

Gene Ontology (GO) analysis comprises three categories, biological process (BP), cellular component (CC), and molecular function (MF), which is important in the exploration of biological functions^43^. Kyoto Encyclopedia of Genes and Genomes (KEGG) is a widely used database that stores information about genomes, biological pathways, diseases, and drugs, and KEGG analysis is more often used to explore potential pathways^44^. Gene Set Enrichment Analysis (GSEA) is a computational method that determines whether a previously identified gene set shows statistically significant differences between two diverse experimental data sets, which helps uncover the collective behavior of genes in states of health and disease^45^. We used the “clusterProfiler” R package^46^ to perform and visualize the GO and KEGG enrichment analyses of the unique DEmRNAs in the DElncRNAs-related networks as well as the GSEA analysis of all the genes of NSCL/P patients and healthy control in GSE183527.

### Patient samples and ethical approval

A total of three sets of paired NSCL/P tissues were obtained from patients undergoing surgical treatment for NSCL/P at the Affiliated Hospital of Stomatology, Nanjing Medical University, between October 2022 and March 2023. These paired tissues included the experimental group tissues were extracted from the NSCL/P-affected regions and the corresponding normal control group tissues, which were collected from adjacent cleft margin areas, typically identified as the relaxed incision area. This study received approval from the Ethical Committee Department, Affiliated Hospital of Stomatology, Nanjing Medical University (Approval number: PJ2022-093-001, 1 October 2022), and before the operation, informed consent was obtained from each patient. We confirm that all methods were performed following relevant guidelines and regulations.

### Animals and tissues collection

Male and female C57BL/6 J mice (n = 12, 6 males, 6 females) were obtained from the Model Animal Research Center of Nanjing University, Nanjing, China (MARC). Female mice (6–8 weeks of age) were housed overnight with males in a 1:1 ratio and checked for vaginal plugs the next morning, which was designated as Day 0.5 of the embryo. Pregnant mice were sacrificed via cervical dislocation at different stages of embryo palatal shelves development: E13.5 (before fusion, approximately 8–9 embryos), E14.5 (period of fusion, approximately 8–9 embryos), and E16.5 (after complete formation, approximately 8–9 embryos) and the palatal shelves were dissected under a microscope and prepared for further experiments. All animals were handled with the approval of the Ethics Committee of the Stomatological School of Nanjing Medical University (Approval number: IACUC-2201021, 19 January 2022). All experiments were performed in conformity with the guidance of the Animal Care Committee of Nanjing Medical University. The study was also conducted according to ARRIVE guidelines.

### Cell culture

The human embryonic palatal mesenchymal (HEPM) cell lines were purchased from American Type Culture Collection (ATCC, Manassas, VA, United States), and cultured in Eagle’s Minimum Essential Medium (ATCC), supplemented with 10% fetal bovine serum (FBS, Gibco), 100 units/ml antibiotics at 37 °C under 5% CO_2_. The human oral keratinocyte (HOK) cell lines were obtained from BeNa Culture Collection (Beijing, China), and cultured in Dulbecco’s Modified Eagle Medium (Gibco) supplemented with 10% FBS (Gibco) and 100 units/ml antibiotics and maintained at 37 °C under 5% CO_2_.

### RNA extraction and real-time quantitative reverse transcription PCR (RT-qPCR)

Total RNA including miRNA from human tissue samples, HEPMs, and HOKs was extracted using RNAiso Plus (Total RNA extraction reagent) (TaKaRa, #9108) according to the manufacturer’s protocol. Complementary deoxyribonucleic acid (cDNA) of total RNA was generated using Hiscript1 III RT SuperMix for qPCR (+ gDNA wiper) (Vazyme, R312-01) The mature miRNA was reverse transcribed by miRNA-specific primers for quantification of *hsa-miR-15b-5p* and *hsa-miR-29c-3p*, *U6* served as a control using the PrimeScript RT reagent kit (TaKaRa, #RR047A). Real-time quantitative reverse transcription PCR (RT-qPCR) reaction was performed using ChamQ SYBR qPCR Master Mix (Vazyme, Nanjing, China) on the ABI-7300 Real-Time PCR System (Applied Biosystems, CA, USA). glyceraldehyde 3-phosphate dehydrogenase (*GAPDH*) and *U6* were used to normalize mRNA and miRNA levels respectively. Gene expression was calculated by the relative expression method (2^−∆∆CT^). The primers used are listed in Table S2.

### ASO and inhibitors design and transfection

The suppression of lncRNA expression was achieved by transfecting antisense oligonucleotides (ASOs) into HEPMs and HOKs. Simultaneously, the downregulation of *hsa-miR-15b-5p* expression was carried out by transfecting inhibitors specific to *hsa-miR-15b-5p*. These ASOs and *hsa-miR-15b-5p* inhibitors were custom-designed and synthesized by GenePharma (Shanghai, China). Transfections were performed using Lipofectamine 2000 reagent (Thermo Fisher Scientific, 11668-019, USA) when cell confluence reached 70–80%. Following transfection, cells were switched to a medium containing 5% serum after six hours and then harvested at 48 h post-transfection. The sequences of the ASOs and *hsa-miR-15b-5p* inhibitors are outlined in Table S2.

### Hematoxylin and eosin (H&E) staining and immunohistochemistry (IHC)

Hematoxylin and eosin (H&E) staining was performed according to the manufacturer’s instructions. In brief, human sample tissues of NSCL/P were immersed in 4% paraformaldehyde for 48 h. Next, the fixed tissues were dehydrated, cleared, and embedded in paraffin wax. The paraffin blocks were cut into 4-μm-thick sections and stained with hematoxylin and eosin (H&E). Primary antibodies against the following proteins were used: EIF3H (1:100, Santa Cruz, sc-271283), SRSF1 (1:100, Santa Cruz, sc-33652), and RBBP6 (1:100, Santa Cruz, sc-9962) (Table S3). Briefly, tissues were fixed, dehydrated, embedded, and sectioned for each sample for all stains. Sections were incubated with primary antibodies, washed, and then incubated with appropriate secondary antibodies (MaxVision, kit-5020, China). Optical microscopy (Thermo Scientific, Wilmington, USA) was used to image stained sections. Semi-quantitative analysis was performed using Fiji v2.9.0 (NIH, Bethesda).

### Immunofluorescence (IF) staining

Murine palatal shelf tissues of E13.5, E14.5, and E16.5 were dewaxed in xylene, and then dehydrated in ethanol. The tissues were then incubated in solutions containing specific primary antibodies: EIF3H (1:100, Santa Cruz, sc-271283), SRSF1 (1:100, Santa Cruz, sc-33652), and RBBP6 (1:100, Santa Cruz, sc-9962) (Table S3) followed by incubation in Alexa fluor 488-labeled goat anti-mouse secondary antibodies (1: 50, Beyotime, A0428).

### Statistical analysis

All experiments were carried out at least three times. All data were expressed as the mean standard error of the mean (S.E.M.). The results in the control and experimental groups were analyzed by GraphPad Prism software (ver.9.5.0, La Jolla, CA). P < 0.05 was considered statistically significant.

### Ethics statement

The studies involving human participants were reviewed and approved by the Ethics Committee Department of Affiliated Hospital of Stomatology, Nanjing Medical University (PJ2022-093-001, 1 October 2022). The patients/participants provided their written informed consent to participate in this study. The animal study protocol was approved by the Ethics Committee of the Stomatological School of Nanjing Medical University (IACUC-2201021, 19 January 2022). All experiments were performed in conformity with the guidance of the Animal Care Committee of Nanjing Medical University.

## Results

### Subsection identification and characterization of differentially expressed lncRNAs associated with non-syndromic cleft lip with or without cleft palate

**Figure 2:**
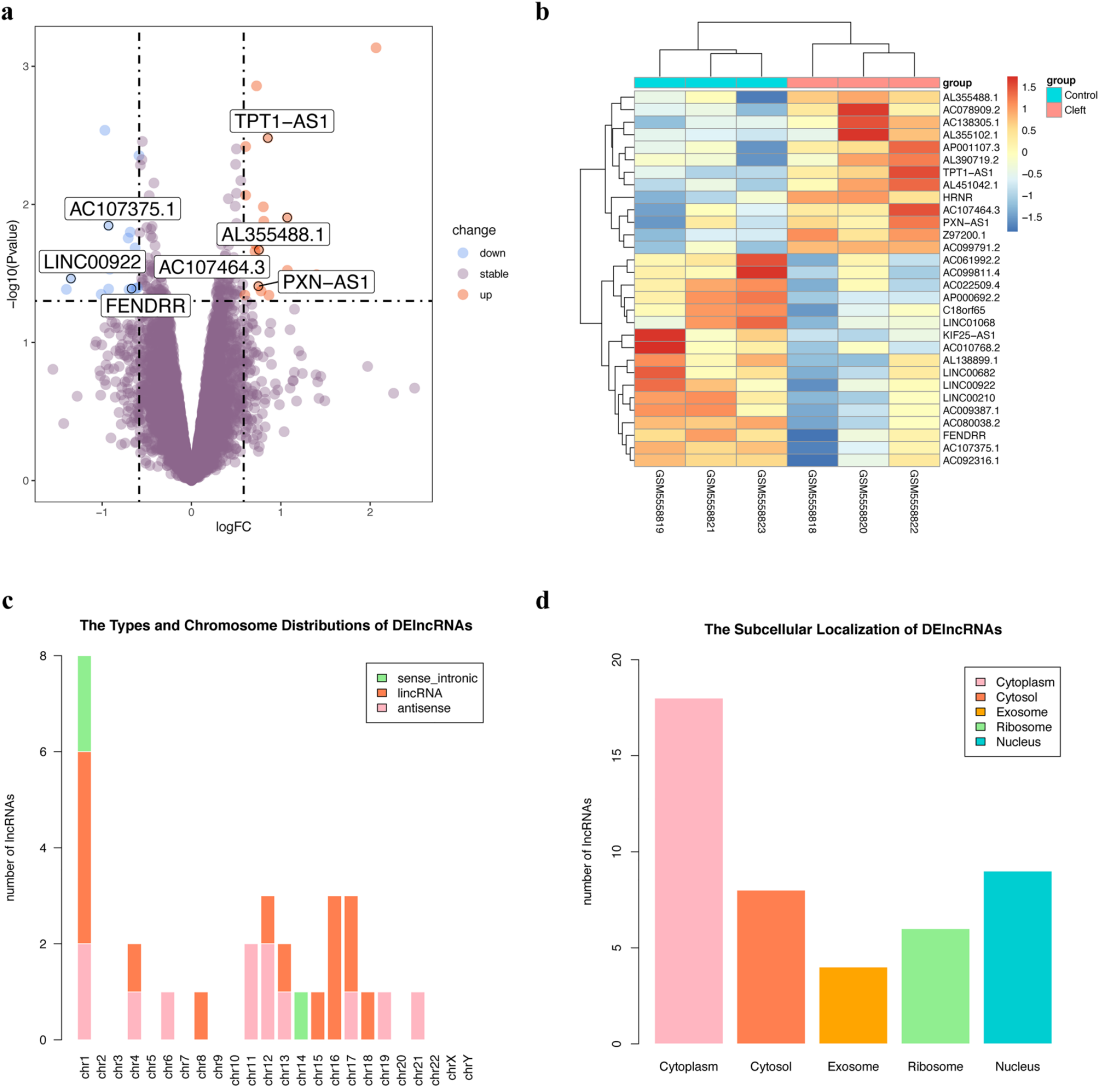
Identification and characterization of differentially expressed lncRNAs associated with non-syndromic cleft lip with or without cleft palate. (**a**) Volcano plot presenting the differentially expressed lncRNAs (DElncRNAs) discovered from the nonsyndromic cleft lip with or without cleft palate (NSCL/P) tissues compared with control samples from the GSE183527 dataset. (**b**) Heatmap showing hierarchical clustering of the expression of the 30 DElncRNAs with a sequence record in the LNCipedia database, which separated NSCL/P from control groups. (**c**) Histograms showed that among the 30 DElncRNAs, most were lincRNAs and were mainly distributed on chromosome 1. (**d**) Histograms showed that most of the 30 DElncRNAs were localized in the cytoplasm.

In this study, we employed the “limma” R package to analyze differentially expressed long non-coding RNAs (DElncRNAs) between trimmed wound edge and adjacent normal tissues of NSCL/P patients of microarray datasets GSE183527. In total, 37 DElncRNAs were identified by screening, of which 19 were downregulated (Fig. 2a, blue dot) and 18 were upregulated genes (Fig. 2a, orange dot). Accordingly, the LNCipedia database was applied to annotate the total DElncRNAs, only 30 of these DElncRNAs possess a sequence record in the LNCipedia database. Then, we used the heat map (Fig. 2b) to show the standardized expression of the 30 DElncRNAs, revealing that hierarchical clustering of the expression of the 30 DElncRNAs can separate NSCL/P from the control group. In addition, these lncRNAs, in the LNCipedia database, were predominantly distributed on chromosome 1 and divided into three categories: sense_intronic (10%), Antisense (40%), and lincRNA (50%) (Fig. 2c). Moreover, the subcellular localization of lncRNAs carries important information for understanding their complex biological functions. Subsequently, we downloaded the sequences of the 30 DElncRNAs from the LNCipedia database to predict the lncRNAs subcellular localizations by the lncLocator database. The results showed that most DElncRNAs were predicted to localize in the cytoplasm (Fig. 2d), while, *TPT1-AS1*, *PXN-AS1*, *FENDRR*, and *KIF25-AS1* were predicted to localize in both the cytoplasm and nucleus. The detailed types, chromosome distributions, and subcellular localizations of these DElncRNAs are displayed in Table S4. These results showed that we identified 30 differentially expressed lncRNAs associated with non-syndromic cleft lip with or without cleft palate and most of them were lincRNA, distributed on chromosome 1, and localized in the cytoplasm.

### Identification of the competing endogenous RNAs (ceRNAs) of cytoplasm-localized DElncRNAs and construction of the competing endogenous RNA network

**Figure 3:**
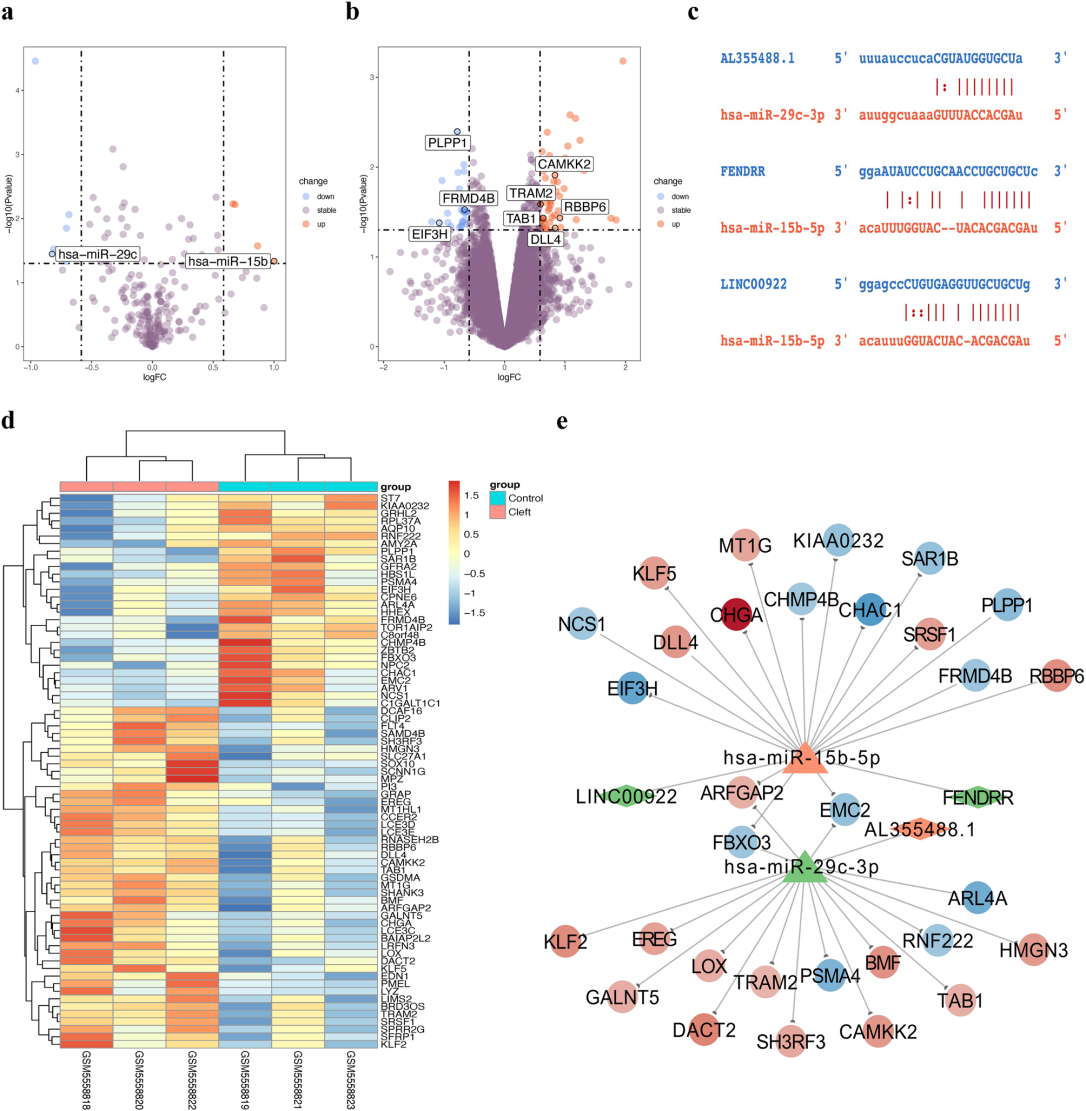
Identification of the competing endogenous RNAs (ceRNAs) of cytoplasm-localized DElncRNAs and construction of the competing endogenous RNA network. (**a**) Volcano plot presenting the differentially expressed miRNAs (DEmiRNAs) discovered from NSCL/P tissues compared with control samples from the GSE47939 dataset. (**b**,**c**) Volcano plot and heat map showing differentially expressed mRNAs (DEmRNAs) discovered from NSCL/P tissues compared with control samples from the GSE183527 dataset. (**d**) Schematic diagram of the putative *has-miR-29c-3p* binding site in the 3′ UTR of *AL355488.1*, and *has-miR-15b-5p* binding site in the 3′ UTR of *FENDRR* and *LINC00922*. (**e**) The ceRNA network in NSCL/P. The circular nodes represent the mRNAs (the orange represents upregulate, and the blue represents downregulate), the triangle nodes represent miRNAs, and the diamond nodes represent the lncRNAs (the orange represents upregulation, and the green represents downregulation).

Several lncRNAs localized in the cytoplasm were found to act as miRNA sponges to regulate gene expression at the translational and post-transcriptional levels. Accordingly, we explored the differentially expressed miRNAs (DEmiRNAs) and mRNAs (DEmRNAs) based on the GSE47939 and GSE183527 datasets to construct a ceRNA network of the DElncRNAs. 12 DEmiRNAs and 73 DEmRNAs were identified by the “limma” package. The volcano plot showed the distribution of DEmiRNAs (Fig. 3a). The distribution of DEmRNAs is shown in Fig. 3b,c. We considered that lncRNAs predicted to localize in other subcellular compartments (cytosol, exosome, and ribosome) were also regarded as cytoplasm-localized lncRNAs, thus, 25 DElncRNAs were selected out. Then, using the ENCORI database, we predicted the targeted miRNAs of the 25 cytoplasm-localized DElncRNAs. The intersection of target miRNAs for cytoplasm-localized DElncRNAs and DEmiRNAs of the GSE47939 dataset was taken. We finally obtained two mature miRNAs: *hsa-miR-29c-3p* (downregulated) and *hsa-miR-15b-5p* (upregulated) of the two pre-miRNAs (*hsa-miR-29c*, *hsa-miR-15b*) in GSE47939 dataset, which interacted with three lncRNAs *AL355488.1* (upregulated), *FENDRR* (downregulated) and *LINC00922* (downregulated) in GSE183527 dataset (Table S5). The 3′ UTR binding location of *hsa-miR-29c-3p* to *AL355488.1* and *hsa-miR-15b-5p* to *FENDRR* and *LINC00922* are shown in Fig. 3d. Subsequently, we applied the R package “multiMiR” to predict the target genes of the two mature miRNAs, and 5761 targets were covered. Followed by, 31 genes were selected which were also the DEmRNAs of the GSE183527 dataset (Table S5). Ultimately, we constructed a ceRNA network including three cytoplasm-localized lncRNAs, two miRNAs, and 31 mRNAs by Cytoscape (Fig. 3e).

### Identification of the interacting RBPs of cytoplasm-localized DElncRNAs and construction of the lncRNA-RBP interaction network

**Figure 4:**
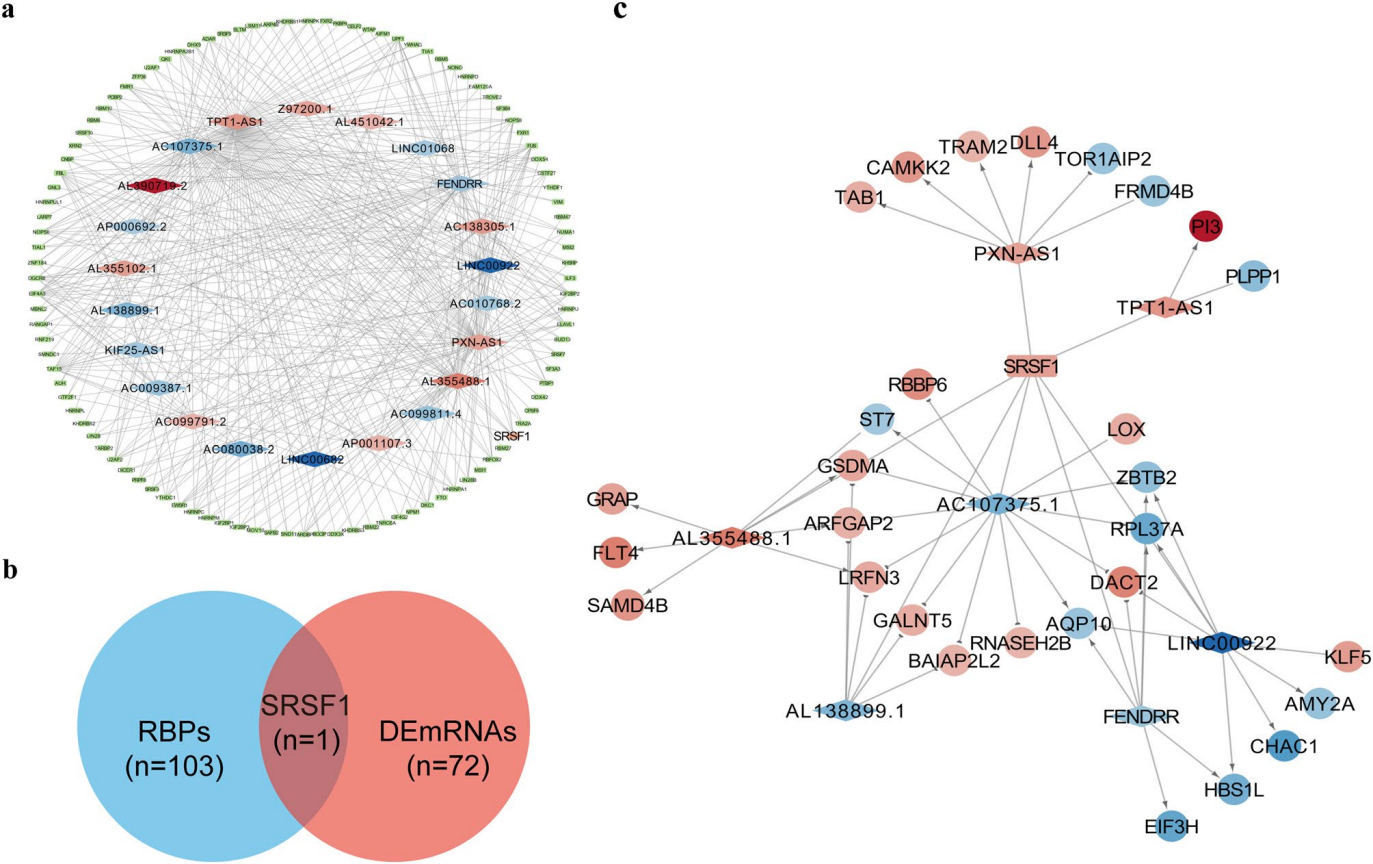
Identification of the interaction RBPs of cytoplasm-localized DElncRNAs and construction of the lncRNA-RBP interaction network. (**a**) The 22 differentially expressed cytoplasm-localized lncRNAs were found to interact with 104 RNA binding proteins (RBPs). Among these, only the RNA-binding protein SRSF1 showed interaction with seven cytoplasm-localized lncRNAs (*TPT1-AS1*, *AL138899.1*, *PXN-AS1*, *AC107375.1*, *LINC00922*, *FENDRR*, and *AL355488.1*) and (**b**) was also identified as a differentially expressed mRNA (DEmRNA) in the GSE183527 dataset. (**c**) The lncRNA-SRSF1 interaction network in NSCL/P. The circular nodes represent the mRNAs, the diamond nodes represent the lncRNAs, the rectangle nodes represent the RNA binding protein (the orange represents upregulation, the blue represents downregulation, the arrow target arrow shape represents positive regulation, the half circle arrow target arrow shape represents negative regulation).

Researches demonstrate that lncRNAs localized in the cytoplasm also can bind with RNA-binding proteins (RBPs), thereby modifying their functions, and reciprocally, influencing downstream gene expression^30,31^. Furthermore, RBPs play a pivotal role in regulating the stability, transport, and localization of lncRNAs^32^. Consequently, we predicted the interaction between 25 cytoplasm-localized DElncRNAs and RBPs using the ENCORI database. From this analysis, we identified 22 cytoplasm-localized DElncRNAs and 104 RBPs (Fig. 4a) and discovered that the RNA-binding protein SRSF1 interacted with seven cytoplasm-localized lncRNAs (*TPT1-AS1*, *AL138899.1*, *PXN-AS1*, *AC107375.1*, *LINC00922*, *FENDRR*, and *AL355488.1*). Importantly, *SRSF1* was also found to be a DEmRNA in the GSE183527 dataset (Fig. 4b). By honing in on SRSF1, an RBP with known significance in diverse cellular processes, including splicing regulation and oncogenesis, we aimed to identify lncRNAs that might exert regulatory effects through their association with this key player. We then used the catRAPID database to predict the ability of SRSF1 to bind with the seven lncRNAs and found that *AC107375.1* had the highest score (Table S6). To further investigate the function of SRSF1 and the seven cytoplasm-localized lncRNAs, we utilized the ENCORI database to predict the target genes of SRSF1. After applying the filter criteria, which included the intersection of target genes and DEmRNAs of the GSE183527 dataset, we obtained 58 mRNAs. Subsequently, we performed Spearman’s correlation analysis to explore the co-expression relationship between the seven cytoplasm-localized lncRNAs and the 58 DEmRNAs. The details of the results are listed in Table S7. We used a cutoff of |r_s_|> 0.9 and P-value < 0.05 to screen for co-expressed lncRNA-mRNA pairs. Finally, we constructed a core lncRNA-RBP interaction network, which included seven cytoplasm-localized lncRNAs, one RBP (SRSF1), and 29 mRNAs (Fig. 4c and Table S8).

### Analysis of cis- and trans-targets of nucleus-localized DElncRNAs and construction of the co-expression network of the lncRNAs and their target genes

**Figure 5:**
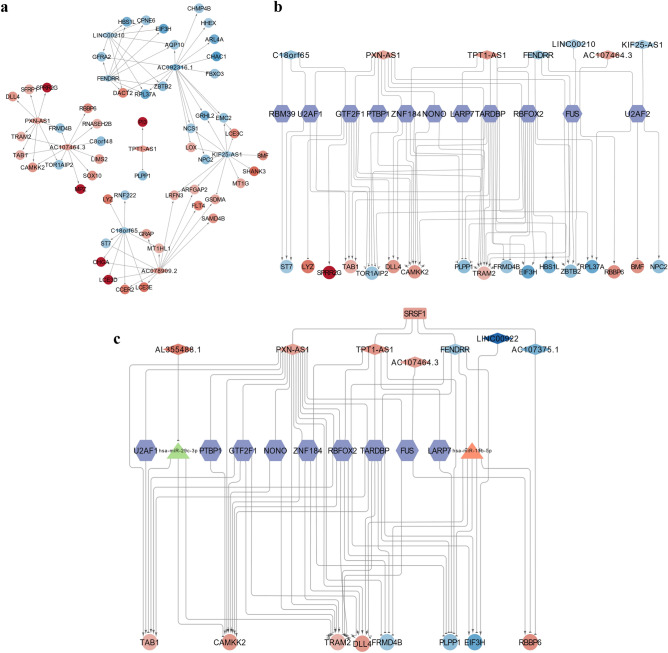
Analysis of trans-targets of nucleus-localized DElncRNAs and construction of the core lncRNA-centered regulatory network in NSCL/P. (**a**) The trans-regulation network in NSCL/P includes 9 nucleus-localized DElncRNAs and 52 DEmRNAs. The circular nodes represent the mRNAs, and the diamond nodes represent the lncRNAs (the orange represents upregulation, the blue represents downregulation). (**b**) The lncRNA-TF interaction network in NSCL/P consists of 7 lncRNAs, 11 transcription factors (TFs), and 17 mRNAs. Most of the TFs were predicted to interact with *PXN-AS1*, *TPT1-AS1*, and *FENDRR*. In addition, most of the mRNAs were predicted to be regulated by TARDBP, PBFOX2, GTF2F1, ZNF184, and FUS. The circular nodes represent the mRNAs, the diamond nodes represent the lncRNAs, and the hexagon nodes represent TFs, the edges between them mean that the lncRNAs potentially interact with the TFs, and positively (the target arrow shape is an arrow) or negatively (the target arrow shape is half circle) regulate the mRNAs. The orange represents upregulation, and the blue represents downregulation. (**c**) The core lncRNA-centered regulatory network in NSCL/P. Seven lncRNAs, one RBP, two miRNAs, nine TFs, and eight mRNAs were included. The diamond nodes represent the lncRNAs, the rectangle node represents RBP, the triangle nodes represent miRNAs, the hexagon nodes represent TFs, and the circular nodes represent the mRNAs. The orange of lncRNAs, RBP, and mRNAs represents upregulation, while the blue represents downregulation. The orange of miRNA represents upregulation, and the green represents downregulation. The arrow target arrow shape represents positive regulation, and the half circle represents negative regulation.

LncRNAs localized in the nucleus were found to regulate gene transcription or pre-transcription levels by binding to the DNA promoter region or transcription factors, namely, homeopathic regulation (cis-regulate) and trans regulation (trans-regulate). LncRNAs can exert their regulatory functions by influencing the expression of target genes, which is often reflected in the correlation of their expression levels. Accordingly, to further investigate the cis- and trans-regulation, we performed Spearman’s correlation analysis to explore the co-expression relationship between nine nucleus-localized DElncRNAs and the 73 total DEmRNAs. The detailed results are displayed in Table S9. We used a cutoff of |r_s_|> 0.8 and P-value < 0.05 to screen for co-expressed lncRNA-mRNA pairs which may conform to the cis-regulatory relationship. Nine lncRNA-mRNA pairs, located on the same chromosome, were filtered out (Table S10). While none of the target mRNAs was located within 100 kb of their paired-lncRNA, we encountered challenges in constructing a lncRNA cis-regulatory network in NSCL/P. In addition, genes with |r_s_|> 0.9 and P-value < 0.05 in the co-expression analysis were used as the trans-regulate target genes of nucleus-localized DElncRNAs, and 52 DEmRNAs met the screening criteria (Table S11). The regulatory relationship between the nine nucleus-localized DElncRNAs and their target DEmRNAs is shown in Fig. 5a. Nevertheless, it is important to note that Spearman’s correlation analysis of DElncRNAs and DEmRNAs does not directly investigate the physical binding of lncRNAs to DNA. Furthermore, studies have indicated that lncRNAs function as molecular scaffolds, facilitating the recruitment of transcription factors (TFs) to the promoter regions of target genes^39^. These lncRNAs play a role in modulating the effects of TFs, exhibiting the ability to either enhance or attenuate their impact, and, in certain instances, even reverse their regulatory actions^18,40^. To provide a more comprehensive understanding of the relationship between lncRNAs and mRNAs, we extended our analysis. Specifically, we obtained TFs significantly correlated with DEmRNAs from the GeneCards database and identified RBPs associated with the corresponding DElncRNAs from the ENCORI database. Notably, we selected TFs that served both as RBPs for the DElncRNAs and as TFs for the DEmRNAs to ensure a more precise delineation of regulatory interactions. A DElncRNAs-trans-regulation network, including seven nucleus-localized DElncRNAs, 11 TFs, and 17 DEmRNAs, was built to explore the potential regulatory functions of nucleus-localized DElncRNAs. As shown in Fig. 5b, most of the nucleus-localized DElncRNAs (*PXN-AS1*, *TPT1-AS1*, *AC107464.3*, *C18orf65*, *FENDRR*, *LINC00210*, and *KIF25-AS1*) participated in pathways regulated by several crucial TFs, including GTF2F1, TARDBP, RBFOX2, ZNF184, FUS, U2AF1, U2AF2, NONO, PTBP1, RBM39, and LARP7. We found that lncRNA may correspond to multiple mRNAs through interacting with different TFs, and one mRNA may correspond to multiple lncRNAs. The relationship between the two is not necessarily one-to-one. Collectively, we constructed a trans-regulatory network of the 7 nucleus-localized DElncRNAs and found 9 lncRNA-mRNA pairs might conform to the cis-regulatory relationship. The analysis may provide useful references for further research.

### Comprehensive analysis of all the DElncRNAs and construction of the core interaction network in NSCL/P

The target mRNAs are crucial for conveying the function of lncRNAs. To further investigate the core regulatory relationship of lncRNAs in NSCL/P among the previously identified three networks (Table S12), we integrated the ceRNA network, lncRNA-SRSF1 interaction network, and trans-regulation network to identify co-target genes. We selected eight co-target genes common to all three networks and constructed a core interaction network (Fig. 5c), which includes seven lncRNAs (*AL355488. 1*, *TPT1-AS1*, *PXN-AS1*, and *AC107464.3* were up-regulated; *LINC00922*, *FENDRR*, and *AC107375.1* were down-regulated), nine TFs (U2AF1, PTBP1, GTF2F1, NONO, ZNF184, RBFOX2, TARDBP, FUS, and LARP7), one RBP (SRSF1 was up-regulated), two miRNAs (*hsa-miR-15b-5p* was up-regulated, and *hsa-miR-29c-3p* was down-regulated) and eight mRNAs (*TAB1*, *CAMKK2*, *TRAM2*, *DLL4*, and *RBBP6* were up-regulated; *FRMD4B*, *PLPP1*, and *EIF3H* were down-regulated). To sum up, we constructed a core interaction network, and further research into this network may lead to the identification of new therapeutic targets for NSCL/P.

### Functional enrichment analysis and gene set enrichment analysis

**Figure 6:**
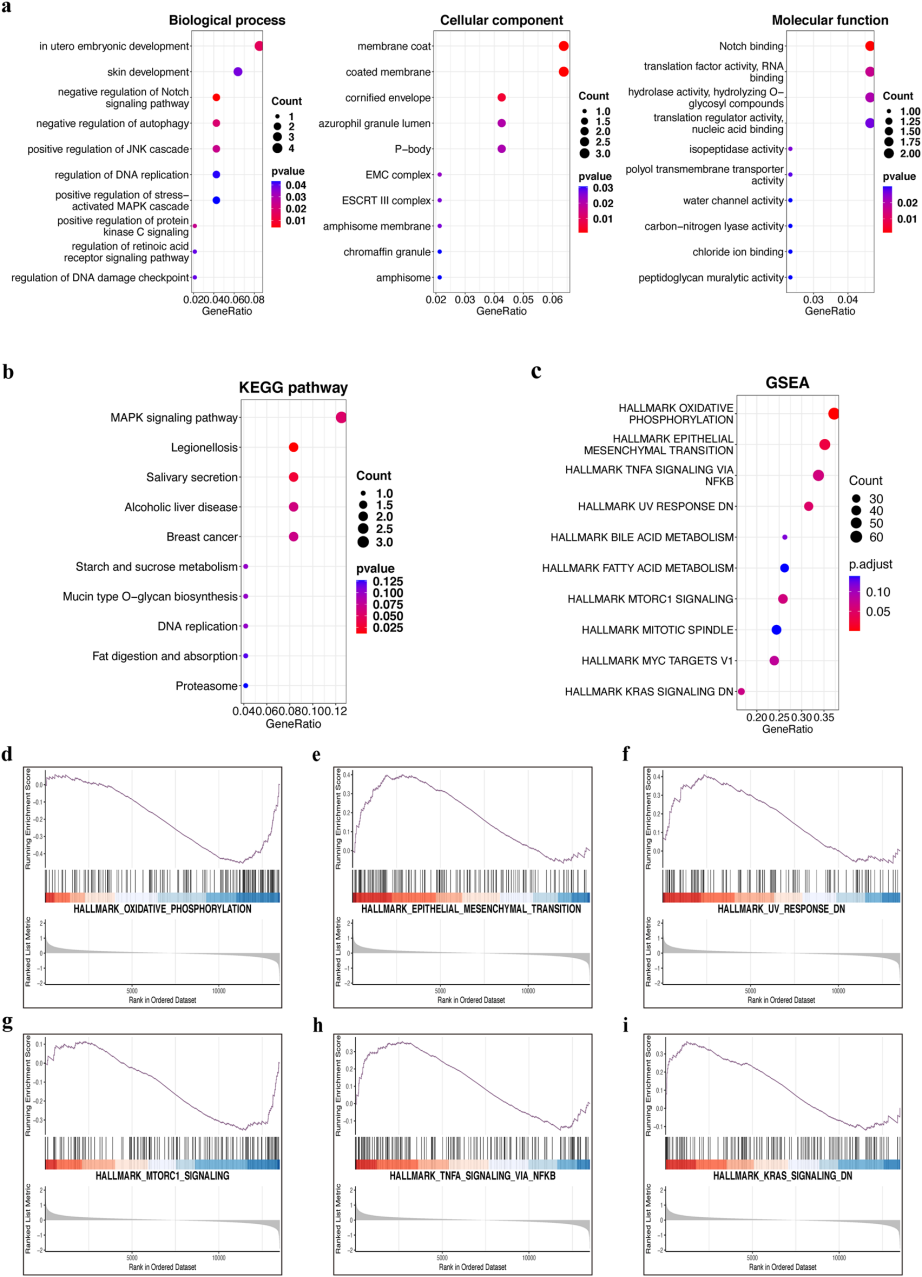
Functional enrichment analysis and gene set enrichment analysis. (**a**) The three parts: biological processes, molecular functions, and cellular components of GO analysis. (**b**) The KEGG signal pathway of DElncRNAs targeted DEmRNAs. (**c**) The top 10 significantly enriched hallmarks of GSEA between NSCL/P and the control group. (**d**–**i**) The oxidative phosphorylation, epithelial-mesenchymal transition (EMT), UV response, MTORC1 signaling, (TNF) α signaling via NF-kB pathways and KRAS signaling may be related to the development of NSCL/P.

The function of the lncRNA can be inferred from the function of target mRNAs. Accordingly, to predict the potential functions of the dysregulated lncRNAs in NSCL/P, we select 49 unique mRNAs (Table S12) from the three lncRNA-associated networks to perform enrichment analysis, including Gene Ontology (GO) and Kyoto Encyclopedia of Genes and Genomes (KEGG) analyses. The GO analysis revealed that the NSCL/P group had complicated functional pathways compared to the controls, containing associated differentially expressed mRNAs with a P-value < 0.05. Figure 6a shows the significantly enriched GO terms. These enriched mRNAs were functionally classified by BP, CC, and MF. Among them, several BPs seemed to be associated with the mechanisms of NSCL/P, for instance, “in utero embryonic development”, “negative regulation of Notch signaling pathway”, “negative regulation of autophagy”, “positive regulation of JNK cascade”, “positive regulation of protein kinase C signaling”, “skin development”, “regulation of retinoic acid receptor signaling pathway”, “regulation of DNA damage checkpoint”, “regulation of DNA replication”, “positive regulation of stress-activated MAPK cascade”. Moreover, many CCs and MFs were found to be related to the regulation of cell proliferation and autophagy, such as “membrane coat”, “P-body”, “EMC complex”, “ESCRT III complex”, “chromaffin granule”, “Notch binding”, “translation factor activity”, “RNA binding”, “isopeptidase activity”. In addition, KEGG analysis revealed significant gene expressions in the MAPK signaling pathway (Fig. 6b). Interestingly, the “DNA replication” and “proteasome” terms were also enriched, which was consistent with the GO analysis.

To avoid the one-sidedness caused by only using the intersection gene enrichment, we also performed Gene set enrichment analysis (GSEA) on all genes of NSCL/P patients in GSE183527. The reference gene set was “h.all.v7.4.symbols.gmt” from the MSigDB collection. We selected the top 10 significantly enriched signaling pathways based on the criteria of FDR < 0.25, NOM P-value < 0.05, and |NES|> 1 (Fig. 6c). The most significantly enriched pathways were “oxidative phosphorylation” (Fig. 6d), “epithelial-mesenchymal transition (EMT)” (Fig. 6e), “UV response” (Fig. 6f), “MTORC1 signaling” (Fig. 6g), “(TNF) α signaling via NF-kB” (Fig. 6h), “KRAS signaling” (Fig. 6i). The GSEA results provide a significant molecular basis for a better understanding of how lncRNAs play a role in NSCL/P.

### RT‑qPCR verification of differentially expressed genes in the NSCL/P human samples and the core lncRNA-centered regulatory network in vitro

**Figure 7:**
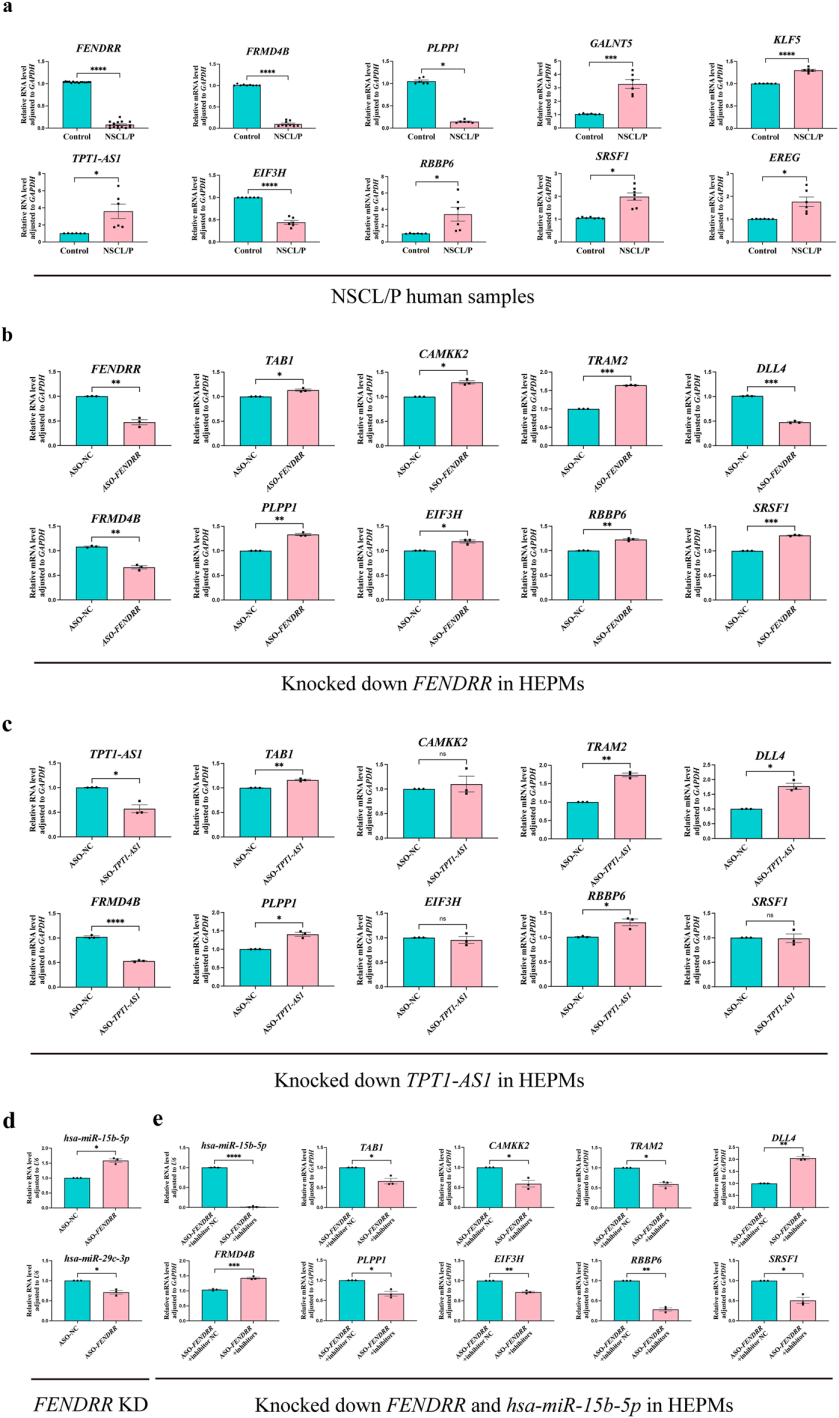
Verification of the mRNA expression of differentially expressed genes in NSCL/P human samples and confirm the core lncRNA-centered regulatory network in vitro. (**a**) RT-qPCR results were consistent with bioinformatics results. (**b**,**c**) RT-qPCR results showed the dysregulated expression of the target genes after knocking down *FENDRR* (**b**) and *TPT1-AS1* (**c**) in the human embryonic palatal mesenchyme (HEPM) cells. (**d**) RT-qPCR results showed the dysregulated expression of *hsa-miR-15b-5p* (up-regulated) and *hsa-miR-29c-3p* (down-regulated) after knocking down *FENDRR*, and (**e**) the expression of target genes was reversed by simultaneously knocking down *FENDRR* and *hsa-miR-15b-5p* in the human embryonic palatal mesenchyme (HEPM) cells. *p < 0.05, **p < 0.01, ***p < 0.001, and ****p < 0.0001; *ns* not significant.

Microarray data is significantly correlated with RT-qPCR results. Subsequently, we selected 15 differentially expressed genes from the lncRNA-related networks, specifically chosen for their utmost relevance to NSCL/P based on insights derived from previous studies. This selection was employed to validate the microarray data results using tissues obtained from three NSCL/P patients who underwent surgical treatment. We found that the expression levels of the following genes were consistent with the sequencing results: *FENDRR*, *EIF3H*, *FRMD4B*, and *PLPP1* (down-regulated); *RBBP6*, *TPT1-AS1*, *SRSF1*, *GALNT5*, *EREG*, and *KLF5* (up-regulated) (Fig. 7a). Additionally, the expression levels of the following genes were incongruent with the sequencing results: *PXN-AS1*, *TAB1*, and *CAMKK2* (down-regulated); *TRAM2* and *DLL4* (no significant difference) (Fig. S3a). These findings demonstrate a substantial concordance (77%) between the RT-qPCR results and our bioinformatics analysis, underscoring the reliability of our computational predictions. Furthermore, to validate the core lncRNA-centered regulatory network (Fig. 5c) in an in vitro setting, we employed antisense oligonucleotides (ASOs) to knock down *FENDRR* and *TPT1-AS1* based on the RT-qPCR results in human embryonic palatal mesenchyme (HEPM) cells and human oral keratinocyte (HOK) cells. Subsequent analysis revealed significant dysregulation of most target genes upon *FENDRR* and *TPT1-AS1* knockdown in HEPMs (Fig. 7b,c) and HOKs (Fig. S3b,c).

Notably, *TPT1-AS1* and *FENDRR* exhibited contrasting expression patterns in NSCL/P human samples, yet the dysregulation pattern of most target genes following the knockdown of both *TPT1-AS1* and *FENDRR* shared a similar expression profile. This suggests a plausible scenario wherein *TPT1-AS1* and *FENDRR* may share common downstream effectors or regulatory pathways. An additional point of interest is the discernible impact of *FENDRR* knockdown on the expression of *TAB1*, *CAMKK2*, and *TRAM2*, which were not initially predicted targets of *FENDRR*. To address this discrepancy, we examined the expression of two miRNAs (*hsa-miR-15b-5p* and *hsa-miR-29c-3p*) in our ceRNA network (Fig. 7d and Fig. S3d). As anticipated, the knockdown of *FENDRR* led to an upregulation of *hsa-miR-15b-5p*, consistent with predictions. Intriguingly, *FENDRR* knockdown also resulted in a decrease in *hsa-miR-29c-3p*, suggesting a potential mechanism for the upregulation of the target genes (*TAB1*, *CAMKK2*, and *TRAM2*) of *hsa-miR-29c-3p*. Despite the prediction that *FENDRR* does not directly regulate *hsa-miR-29c-3p*, these results hint at a potential overlapping effect between them.

Furthermore, to deepen our understanding of the interplay within the *FENDRR*-*hsa-miR-15p-5p* axis and its impact on target expression, we simultaneously knocked down both *FENDRR* and *hsa-miR-15p-5p* in HEPMs and HOKs. Notably, we observed a reversal in the expression of target genes after the dual knockdown compared with the knockdown of *FENDRR* alone (Fig. 7e and Fig. S3e). Additionally, following the ceRNA hypothesis, target genes were expected to exhibit a similar expression pattern as *FENDRR*. However, after *FENDRR* knockdown, the expression of *PLPP1*, *EIF3H*, *RBBP6*, and *SRSF1* increased. This discrepancy may be attributed to the regulation of these genes by other lncRNAs. Consequently, the sole knockdown of *FENDRR* might not only fail to reduce the expression of these targets but could also trigger compensatory mechanisms leading to the upregulation of these genes. In conclusion, these findings suggest that *FENDRR* and *TPT1-AS1* may potentially play roles in the development of NSCL/P, warranting further comprehensive investigation into their functions.

### Protein expression profiling validates key genes in NSCL/P pathogenesis

**Figure 8:**
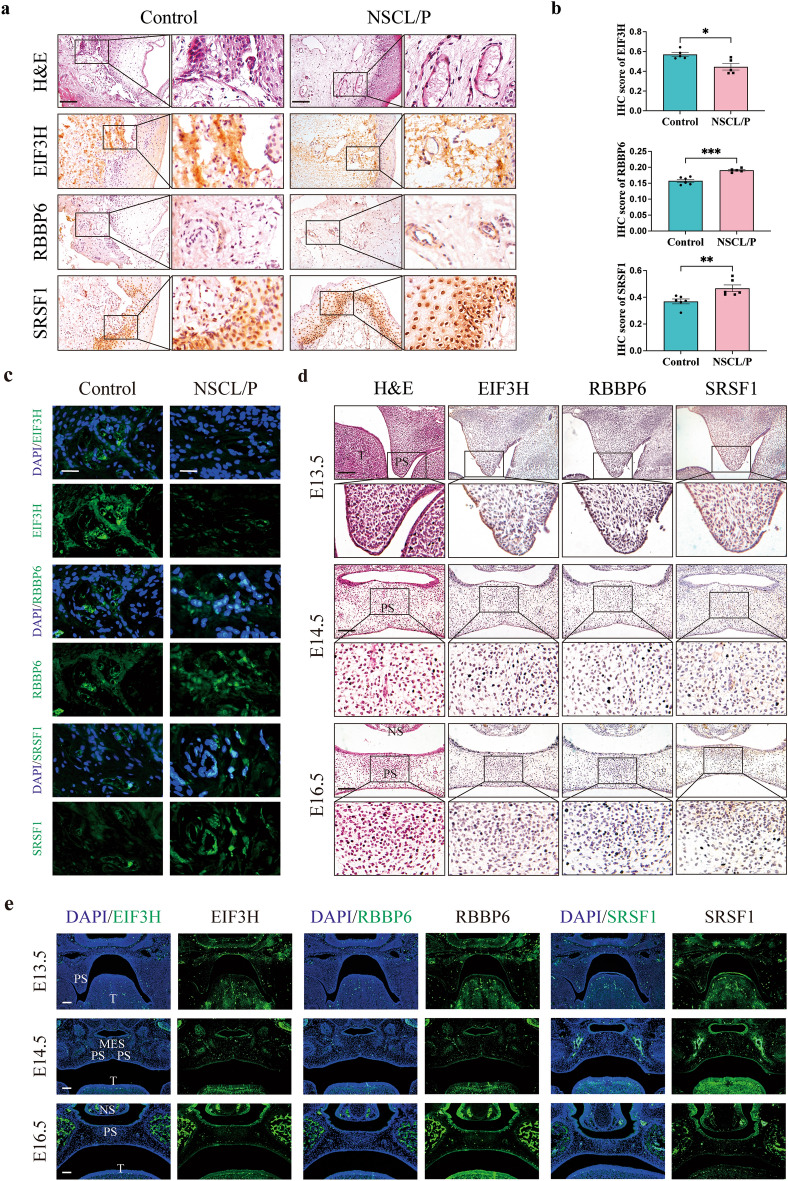
Verification of the protein expression of EIF3H, RBBP6, and SRSF1 in NSCL/P tissues and wild-type murine palatal shelves. (**a**) Hematoxylin and eosin (H&E) staining, immunohistochemical staining, and (**b**) semi-quantitative data showing the relative expression of EIF3H, RBBP6, and SRSF1. Scale bar 100 μm. (**c**) Representative fluorescence images of EIF3H, RBBP6, and SRSF1 (green) in NSCL/P tissues and controls. Scale bar 25 μm. (**d**) Hematoxylin and eosin (H&E) staining, immunohistochemical staining, and (**e**) (IF) showing the relative expression of Eif3h, Rbbp6, and Srsf1 in the developing palatal shelves. *p < 0.05, **p < 0.01, ***p < 0.001, and ****p < 0.0001; *ns* not significant; scale bar 100 μm; *PS* palatal shelf, *T* tongue, *NS* nasal septum, *MES* medial edge epithelial seam.

Drawing on prior research^47–53^ and guided by RT-qPCR results, we selected three differentially expressed genes (DEGs)—*EIF3H*, *SRSF1*, and *RBBP6*—for in-depth exploration. These genes were selected based on their documented roles in the regulation of critical cellular processes, including but not limited to cell proliferation, differentiation, adhesion, migration, apoptosis, and epithelial-mesenchymal transition (EMT), which are believed to be pivotal in the context of NSCL/P. To assess the expression of these genes at the protein level, we conducted immunohistochemistry (IHC) and immunofluorescence (IF) analyses in NSCL/P human samples. As illustrated in Fig. 8a–c, these analyses were consistent with the RT-qPCR results. Moreover, we expanded our investigation to murine models, frequently employed for studying craniofacial morphogenesis due to the similarity of their developmental processes to those of humans, occurring within a condensed timeframe. Consequently, we evaluated protein expression in murine palatal shelves collected at E13.5 (before fusion), E14.5 (period of fusion), and E16.5 (after complete formation) via IHC and IF. Notably, our observations revealed positive protein expression in the mesenchyme and some regions of the ectoderm within the developing palatal shelves (Fig. 8d,e). At E13.5 and E14.5, the activity of EIF3H, RBBP6, and SRSF1 was observed widely distributed in palatal mesenchyme and tongue. Moreover, the expression became concentrated at the medial edge epithelial seam (MES) at E14.5. Moving forward to E16.5, following the fusion period, a reduction in their expression was noted in the palatal region, while an increase was observed in the maxillary bone. These robust findings may not only validate the credibility of our bioinformatics analyses but also might offer valuable insights into unraveling the pathogenesis of NSCL/P.

## Discussion

Non-syndromic cleft lip with or without cleft palate (NSCL/P) is a common congenital craniofacial anomaly that results from the incomplete fusion of the lip and/or palate during embryonic development. Previous research has identified several genetic and environmental factors that contribute to NSCL/P, but the specific molecular mechanisms underlying the disorder remain unclear. The identification of differentially expressed genes and pathways in this study provides new insights into the pathogenesis of NSCL/P. In this study, we identified several lncRNAs (*AL355488.1*, *FENDRR*, *LINC00922*, *TPT1-AS1*, *PXN-AS1*, *AC107464.3*, *AC107375.1*, *C18orf65*, *LINC00210*, *KIF25-AS1*, and *AL138899.1*), which had all been rarely reported in NSCL/P and not the causative genes for cleft lip with or without cleft palate (CL/P), Moreover, we preliminarily substantiated the regulatory roles of lncRNAs *FENDRR* and *TPT1-AS1* in HEPM cells and HOK cells. Furthermore, we verified the protein expression levels of three chosen genes (*EIF3H*, *RBBP6*, and *SRSF1*), whose functions, based on previous research, are more likely to be linked to NSCL/P development. These validations were accomplished through IHC and IF analyses in both human samples and murine palatal shelves.

Research on the role of lncRNAs in NSCL/P has gained increasing attention in recent years. Several studies have identified dysregulated lncRNAs in NSCL/P and their impact on disease development^9–12^. Mechanistic insights into lncRNA involvement in NSCL/P have been explored through the construction of competing endogenous RNA (ceRNA) networks. For instance, one study highlighted the potential regulatory role of *NONMMUT004850.2*/*NONMMUT024276.2*-*miR-741-3p*/*miR-465b-5p*-*Prkar1α* in palatal fusion during cleft palate development^54^. Another investigation revealed a complex regulatory association involving *miR-483-3p*, *miR-4690-3p*, *miR-654-3p*, *miR-6515-5p*, lncRNA *RP11-731F5.2*, lncRNA *XIST*, lncRNA *RP11-591C20.9*, *RARA*, and *SMPD1* in the CL/P and CPO groups^55^. Additionally, a ceRNA regulatory network was elucidated, where LncRNA-*NONMMUT100923.1* regulates *Cdsn* expression by competitively binding to endogenous *miR-200a-3p* during palatogenesis in an all-trans retinoic acid (ATRA) induced murine model^56^. Some studies utilizing the same lncRNA dataset GSE183527 as ours have identified ceRNA networks potentially contributing to the etiology of non-syndromic orofacial clefts (NSOFC). These networks include *MALAT1*-*hsa-miR-1224-3p*-*SP1*, *MALAT1*-*hsa-miR-6734-5p*/*hsa-miR-1224-3p*-*WNT10A*, *NEAT1*-*hsa-miR-140-3p.1*-*CXCR4*, *NEAT1*-*hsa-miR-3129-5p*/*hsa-miR-199a-3p*/*hsa-miR-199b-3p*-*ZEB1*^10^, and *NEAT1*-*hsa-miR-130b-3p*/*hsa-miR-212–3p*/*hsa-miR-200b-3p*-*SMAD2*^57^. Compared with previous studies, our study enriched and constructed several comprehensive lncRNA-centered regulatory networks by considering the specific role based on their subcellular localization. We presented not only a ceRNA network, but also a lncRNA-SRSF1 interaction network, and a lncRNA trans-regulation network in NSCL/P, which, to our knowledge, have not been reported before.

Our study identified two lncRNAs (*FENDRR* and *TPT1-AS1*) that may contribute to the development of NSCL/P. Insights from gene targeting experiments have unveiled that the absence of *Fendrr* results in compromised differentiation of tissues originating from the lateral mesoderm, specifically affecting the heart and body wall. This developmental impairment culminates in mouse embryonic lethality, typically occurring between E12.5 and E14.5^58^. This pivotal timeframe aligns with the crucial process of palatal fusion, suggesting a potential influence of *Fendrr* on this critical developmental event. It is likely to have an etiological overlap between NSCL/P and cancer^59^, indicating that *FENDRR* might play similar roles in NSCL/P. One study demonstrated that *FENDRR* could inhibit cervical cancer proliferation and invasion by targeting *miR-15a/b-5p* and regulating *TUBA1A* expression^60^. The mechanism was accordant with our ceRNA network results that *FENDRR* acts as an *hsa-miR-15b-5p* sponge to regulate several mRNAs, such as *EIF3H*, *FRMD4B*, *PLPP1*, *RBBP6*, *SRSF1*, and *KLF5* which were dysregulated after knocking down the expression of *FENDRR* in HEPMs and HOKs in our verification. A previous study has shown that *FENDRR* could interact with SRSF1 in Kato III cells to regulate alternative splicing of *MST1R* and induce apoptosis in gastric cancer^61^. Our lncRNA-SRSF1 interaction network also predicted its interaction with SRSF1 to regulate *EIF3H* expression in NSCL/P. In summary, *FENDRR* is closely associated with cell functions and embryonic development. Moreover, the reported regulatory mechanisms align with the networks we screened, indicating a significant correlation between *FENDRR* and NSCL/P.

LncRNA *TPT1-AS1* has been reported to modulate numerous biological processes through multiple mechanisms, including cell proliferation, apoptosis, autophagy, invasion, migration, and epithelial-mesenchymal transition (EMT)^62^, which are known to be involved in NSCL/P progression. Moreover, *TPT1-AS1* functions as a positive regulator of *VEGFA* by binding to NF90, an RNA-binding protein that can upregulate the stability of mRNA, and promotes the interaction between NF90 and *VEGFA* mRNA in colorectal cancer^63^. In our study, *PLPP1* was significantly upregulated after downregulating the expression of *TPT1-AS1* both in HEPMs and HOKs. The above evidence supports the regulatory relationship in the screened lncRNA-SRSF1 network and we speculate that *TPT1-AS1* might interacted with SRSF1 to regulate the mRNA stability of *PLPP1* in NSCL/P.

LncRNA *TPT1-AS1* has been reported to modulate numerous biological processes through multiple mechanisms, including cell proliferation, apoptosis, autophagy, invasion, migration, and epithelial-mesenchymal transition (EMT)^62^, which are known to be involved in NSCL/P progression. Moreover, *TPT1-AS1* functions as a positive regulator of *VEGFA* by binding to NF90, an RNA-binding protein that can upregulate the stability of mRNA, and promotes the interaction between NF90 and *VEGFA* mRNA in colorectal cancer^63^. In our study, *PLPP1* was significantly upregulated after downregulating the expression of *TPT1-AS1* both in HEPMs and HOKs. The above evidence supports the regulatory relationship in the screened lncRNA-SRSF1 network and we speculate that *TPT1-AS1* might interact with SRSF1 to regulate the mRNA stability of *PLPP1* in NSCL/P.

The functions of mRNAs regulated by *FENDRR* and *TPT1-AS1* may reveal their potential significance in NSCL/P. Studies have shown that the eukaryotic translation initiation factor 3H subunit (*EIF3H*), as a deubiquitinase, has a tumor-promoting function via the Wnt/β-catenin signaling pathway in intrahepatic cholangiocarcinoma cells^47^, and promotes Snail-mediated EMT process in esophageal squamous cell carcinoma^48^. Beyond its role in cancer, *EIF3H* has garnered interest as a candidate gene associated with Microcephaly-Thin Corpus Callosum syndrome, highlighting its potential relevance to maxillofacial developmental processes^64^. Intriguingly, studies in mice have revealed that homozygous mutants for the eIF3h^MommeD^^12^ and eIF3h^MommeD^^38^ mutations experience embryonic lethality at E9.5, emphasizing its vital role during early embryonic development^65^. Moreover, in zebrafish embryogenesis, *eif3h* has been found to play a pivotal role in the development of various organs, including the brain, heart, vasculature, and lateral line^66^. Retinoblastoma binding protein 6 (*RBBP6*) is a ubiquitin ligase, which was reported to regulate the ubiquitination of two cell homeostasis-related proteins, YB-1 and p53^49–51^. In addition, *RBBP6* was also reported as a cancer-related protein that has been implicated in the regulation of the cell cycle and apoptosis through the JNK signaling pathway^52^. During human embryonic development, *RBBP6* emerges as an “early riser” with its presence in the oocyte and significant upregulation at 2–8-cell stages. This underscores its critical role in early embryogenesis. Remarkably, functional deficiencies in *RBBP6* have been linked to widespread apoptosis in mouse embryos, leading to embryonic lethality at E7.5^67^. Serine/arginine splicing factor 1 (*SRSF1*) is the archetype member of the SR protein family of splicing regulators. It regulates complex biological pathways, such as mRNA splicing, stability, and translation, as well as other mRNA-independent processes, such as miRNA processing, protein sumoylation, and the nucleolar stress response^53^. The significance of *SRSF1* is further highlighted by its essential role in embryonic development, as *SRSF1* null mice are embryonic lethal. Furthermore, its overexpression has been linked to oncogenic transformation in both rodent and human cells^68^. Particularly relevant to NSCL/P, *SRSF1* has been identified as a direct transcriptional target of MYC, a gene implicated as a probable target effect gene in the 8q24 region associated with NSCL/P^69^.

In previous investigations related to non-syndromic orofacial clefts (NSOFC), a diverse array of tissues has been employed for research endeavors. When comparing normal and experimental groups with the same tissue location, one study opted for dental pulp stem cells due to their shared origin from the neural crest cells^59^, which are also the precursors of lip and palate tissues. In another study, researchers focused on oral keratinocytes from individuals with and without orofacial clefts (OFC)^70^. Their rationale was rooted in the hypothesis that the dysregulation of crucial cellular processes such as proliferation, differentiation, adhesion, migration, and apoptosis in oral keratinocytes contributes to OFC pathogenesis. Conversely, when distinct tissue locations were chosen for the normal and experimental groups, it was commonplace to select tissue adjacent to the cleft margin excised during surgical procedures as the experimental group. Regarding the choice of control tissues, one study utilized umbilical cord samples from mothers of NSCL/P patients^71^. The selection was justified by the shared embryonic origin of the umbilical cord and its involvement in early developmental processes. In another study, normal tissue was collected from healthy individuals during trauma surgeries^11^. In pursuit of a more robust and consistent approach, another study opted for tissues adjacent to the fissure lesion from NSCL/P patients as a self-control^10^. We regarded the latter approach for tissue selection as more appropriate and congruent with our research objectives. Consequently, we curated datasets and obtained human tissue samples using comparable methodologies, thereby indicating the reliability of our study.

There are still limitations to the study, such as the small sample size, the lack of functional validation of the identified lncRNAs and the lncRNA-related networks, and the need for empirical evidence to establish the direct relevance of our findings to NSCL/P. Future studies could focus on validating the function of the identified lncRNAs in NSCL/P pathogenesis using animal models or cell culture systems. Moreover, we encountered challenges in confirming the other lncRNAs (*LINC00922*, *AL355488.1*, *AC107464.3*, *AC107375.1*) expression levels in tissues and assess their concordance with bioinformatics results due to their considerable length, which made it difficult to design specific primers for RT-qPCR assays. In addition, further investigation into the regulatory mechanisms of these lncRNAs and their interactions with other factors, such as environmental exposures, could provide additional insights into the development of NSCL/P. Another important limitation arises from the potential tissue heterogeneity in our collected samples. One noteworthy aspect is that NSCL/P encompasses a spectrum of orofacial cleft types, including cleft lip (CL), cleft palate (CP), and combinations of these. As such, there may be differences in the gene expression patterns of patients with distinct types of clefts. Although our study attempted to address this challenge by obtaining tissues from the same cleft margin for the experimental and control groups, variations in the cellular composition of the tissues could exist. These differences, arising from variations in the type of orofacial cleft, might impact the observed gene expression patterns. Furthermore, an essential consideration is the association between gene expression patterns and cell phenotype modifications. Changes in gene expression do not always directly correlate with cellular phenotype alterations. It is conceivable that certain genes, while differentially expressed, might not have substantial functional consequences in terms of cell behavior. Therefore, we acknowledge the importance of performing additional studies that directly explore the functional implications of the observed gene expression changes.

In summary, this study extends previous research by not only identifying differentially expressed lncRNAs and mRNAs associated with NSCL/P but also providing a more comprehensive analysis of their interactions and regulatory networks. Furthermore, employing RT-qPCR, IHC, and IF, we have preliminarily substantiated the potential roles of lncRNAs *FENDRR* and *TPT1-AS1*, along with mRNAs *EIF3H*, *RBBP6*, and *SRSF1*, in NSCL/P development. However, it is essential to emphasize that we do not posit that the dysregulation of these genes, as described in this study, solely accounts for the pathogenesis of NSCL/P. Instead, we believe that they are integral components of the multifaceted mechanisms underlying NSCL/P, a congenital disorder with a complex etiology. In conclusion, the transcriptional landscape and the identification of associated lncRNAs and critical subnetworks of NSCL/P provided novel insights into the molecular mechanisms in NSCL/P progression, which may pave the way for uncovering the pathogenesis and laying the foundation for future research into the potential regulatory mechanisms of lncRNAs and mRNAs in NSCL/P.

### Supplementary Information

Supplementary Information.

## Data Availability

The datasets analyzed during the current study are available in GEO database (GSE183527, GSE47939, http://www.ncbi.nlm.nih.gov/geo).
