# Supplementary material for: Unveiling dysregulated lncRNAs and networks in non-syndromic cleft lip with or without cleft palate pathogenesis
Source: Sci Rep. 2024 Jan 10;14:1047. doi: 10.1038/s41598-024-51747-8 (PMC10781966; doi:10.1038/s41598-024-51747-8)
Supplement: Supplementary file 1 — Supplementary Information. [file 41598_2024_51747_MOESM1_ESM.docx]

**Supplementary Material**

**Unveiling Dysregulated lncRNAs and Networks in Non-syndromic Cleft Lip with or without cleft palate Pathogenesis**

Caihong Wu^1,2,3^, Haojie Liu^1,2,3^, Zhuorong Zhan^1,2^, Xinyu Zhang^1,2^, Mengnan Zhang^2^, Jiawen You^1,2^, Junqing Ma^1,2,🖂^

^1^ Jiangsu Key Laboratory of Oral Diseases, Nanjing Medical University, Nanjing, China

^2^ Department of Orthodontics, Afﬁliated Hospital of Stomatology, Nanjing Medical University, Nanjing, China

^3^ These authors contributed equally: Caihong Wu and Haojie Liu.

^🖂^Correspondence: Junqing Ma; E-mail: [jma@njmu.edu.cn](mailto:jma@njmu.edu.cn)

**Supplementary Figures and Tables**

## Supplementary Figures


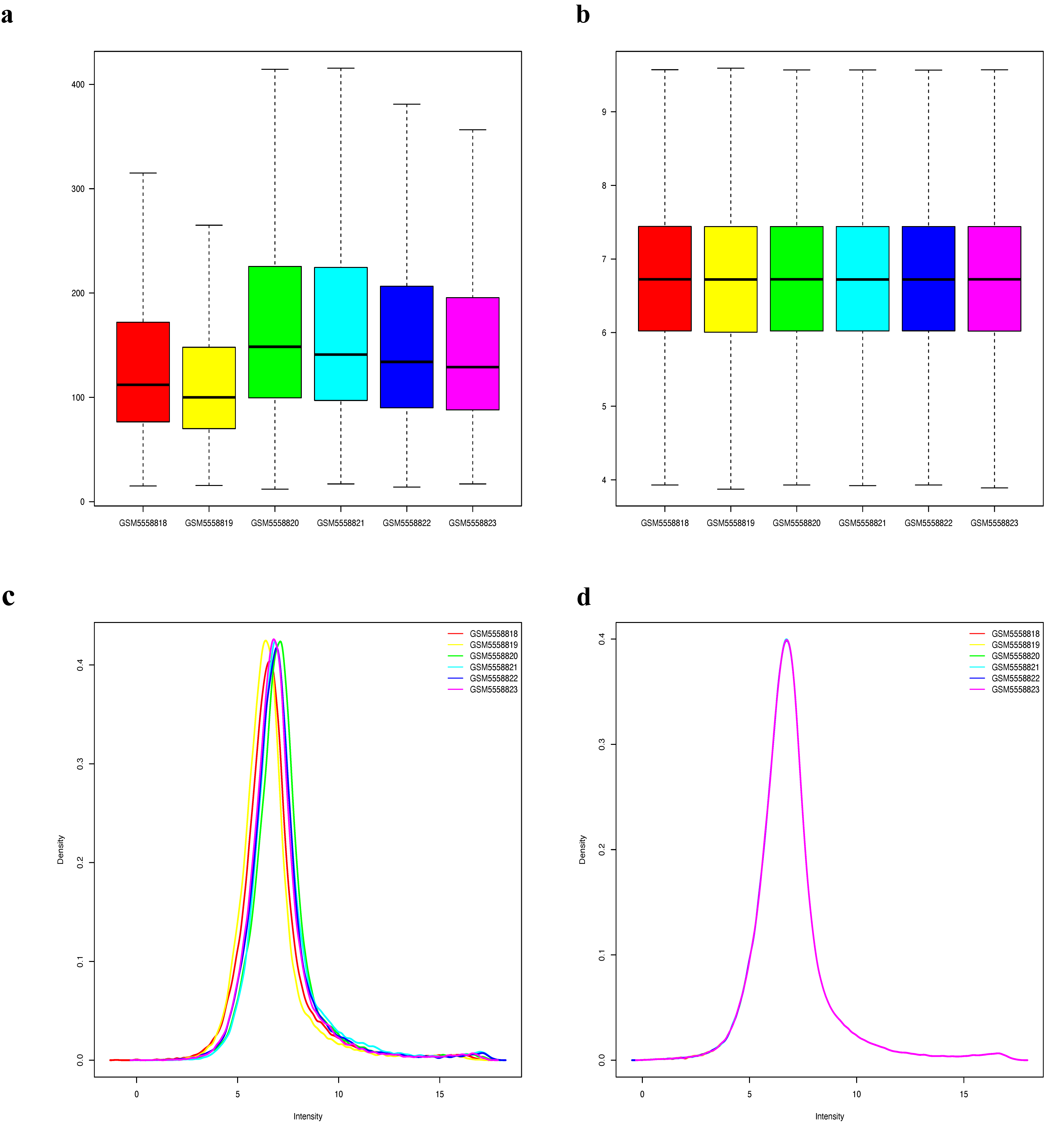


**Figure S1.** Data process of the lncRNA and mRNA dataset GSE183527. **(a, c)** Box plot and density distribution curves presenting the distribution of the raw data before background corrected and normalized. **(b, d)** Box plot and density distribution curves presenting the distribution of the raw data after background corrected and normalized, revealing that the raw data was standardized for further analysis.

**
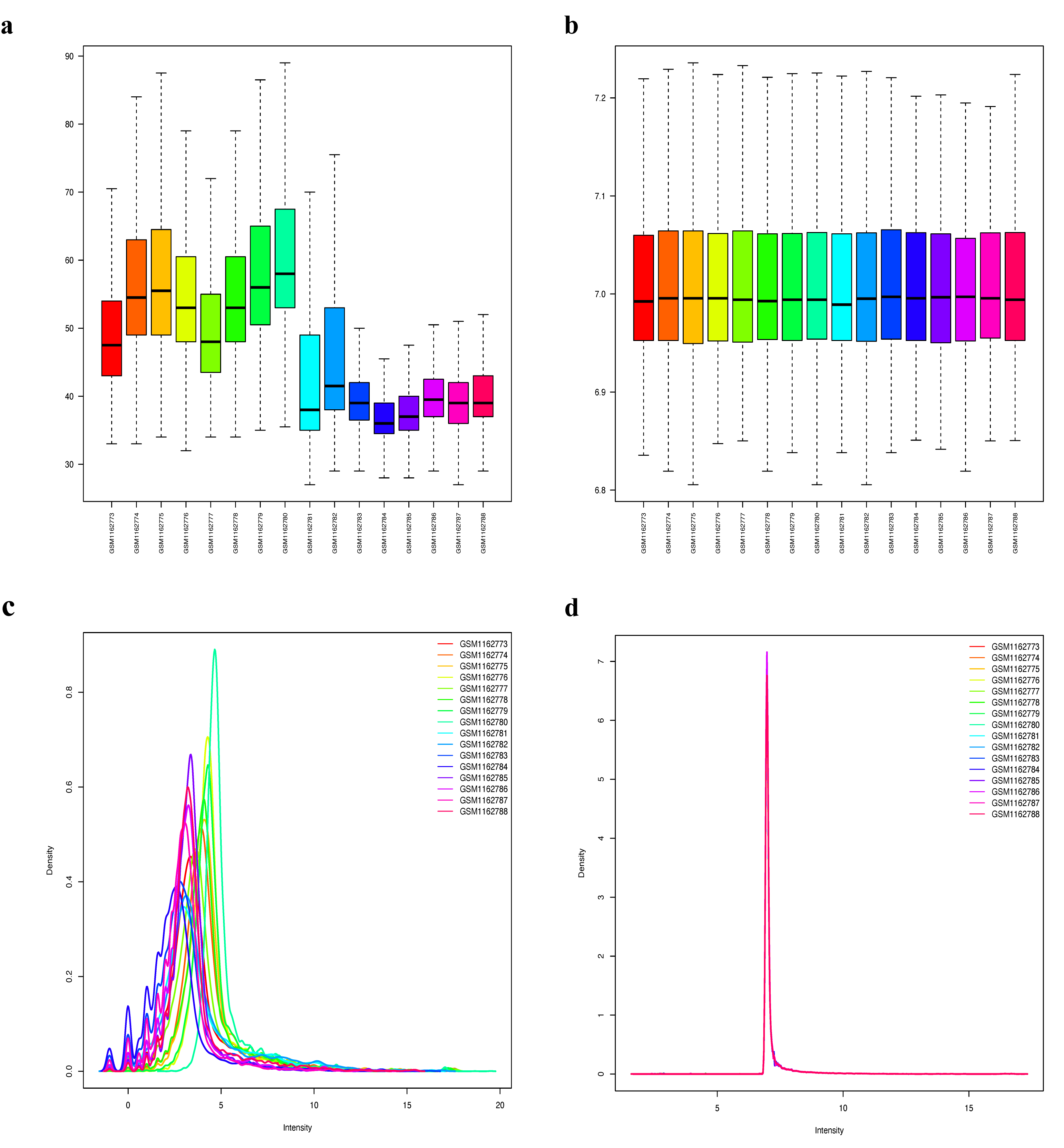
**

**Figure S2.** Data process of the miRNA dataset GSE47939. **(a, c)** Box plot and density distribution curves presenting the distribution of the raw data before background corrected and normalized. **(b, d)** Box plot and density distribution curves presenting the distribution of the raw data after background corrected and normalized, revealing that the raw data was standardized for further analysis.

##
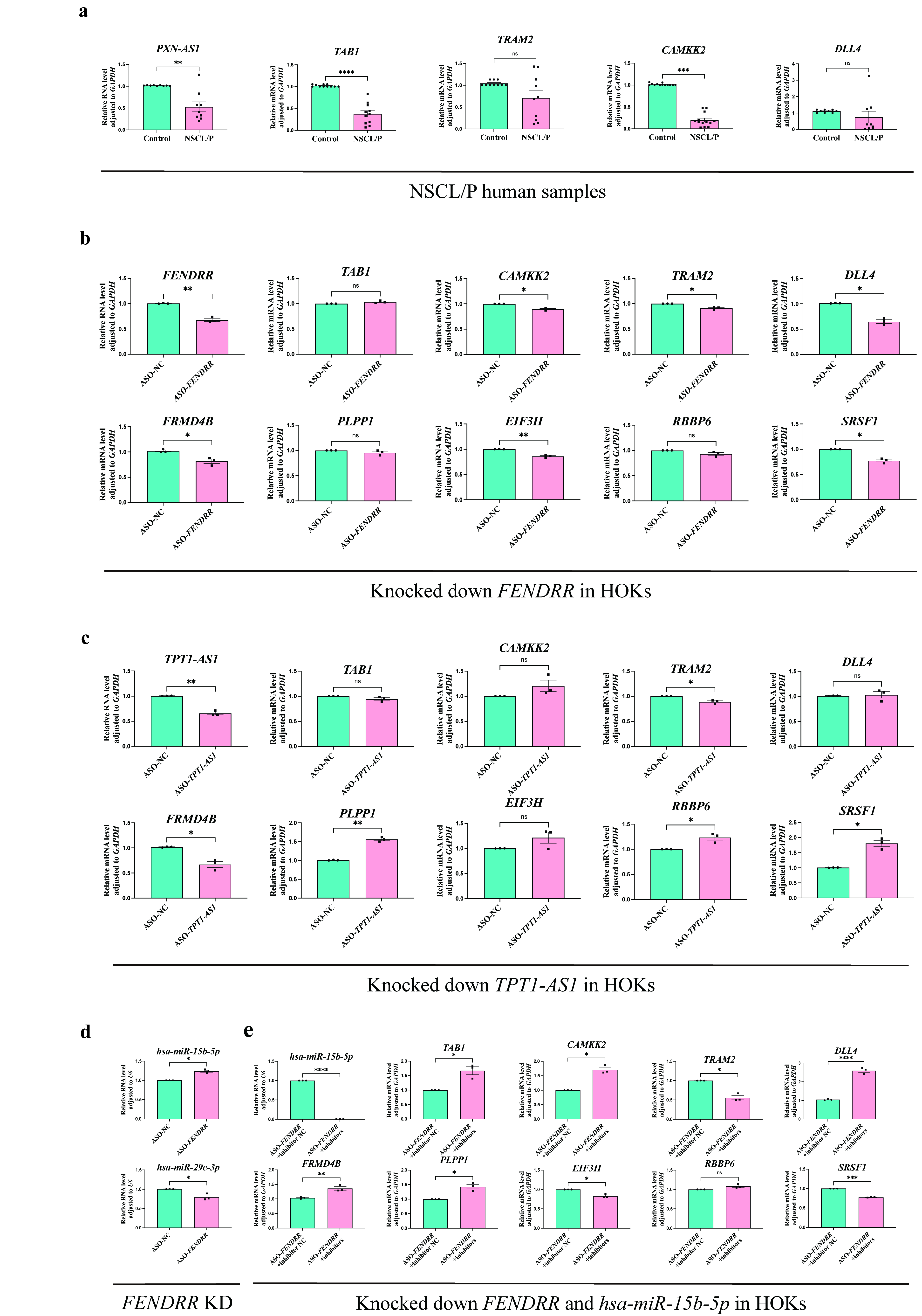


**Figure S3.** Verification of the mRNA expression of differentially expressed genes in NSCL/P human samples and confirm the core lncRNA-centered regulatory network in the human oral keratinocyte (HOK) cells. **(a)** RT-qPCR results were inconsistent with bioinformatics results. **(b-c)** RT-qPCR results showed that the dysregulated expression of the target genes after knocking down *FENDRR* **(b)** and *TPT1-AS1* **(c)** in HOKs. **(d)** RT-qPCR results showed the dysregulated expression of *hsa-miR-15b-5p* (up-regulated) and *hsa-miR-29c-3p* (down-regulated) after knocking down FENDRR, and **(e)** the expression of target genes was in large parts reversed by simultaneously knocking down *FENDRR* and *hsa-miR-15b-5p* in the HOK cells. * p < 0.05, ** p < 0.01, *** p < 0.001, and **** p < 0.0001; ns, not significant.

## Supplementary Tables

**Table S1.** The list of lncRNA/mRNA and miRNA datasets included in this study.

| Datasets | GEO ID | Platform | Samples (number) |
| --- | --- | --- | --- |
| lncRNA/mRNA | GSE183527 | GPL26963  (Agilent-085982 Arraystar human lncRNA V5 microarray) | Cleft palate only (1);  Cleft lip with cleft palate (1);  Cleft lip without cleft palate (1);  Adjacent normal tissue (3) |
| miRNA | GSE47939 | GPL11487  (Agilent-021827 Human miRNA Microarray) | NSCP (10);  Normal control (6) |

GEO: Gene Expression Omnibus; NSCP: non-syndromic cleft lip with cleft palate

**Table S2.** Primer sequences used in this study.

| Primer name | Sequence (5’-3’) | |
| --- | --- | --- |
| Homo-*GAPDH* | F | GGCATGGCCTTCCGTGTCCCC |
|  | R | CACCCTGTTGCTGTAGCCAAATTC |
| Homo-*TAB1* | F | CAAAGCCCGACCTTAACCCT |
|  | R | CCACATAGGGCTCAACACGA |
| Homo-*CAMKK2* | F | TCCAGACCAGCCCGACATAG |
|  | R | CAGGGGTGCAGCTTGATTTC |
| Homo-*TRAM2* | F | TTCCGCAGGAGGACGAAAAG |
|  | R | ACCTCGAACATAAGCCCGATG |
| Homo-*DLL4* | F | AGCTGTAAGGACCAGGAG |
|  | R | ACATTCACAAGCATAGTTGG |
| Homo-*FRMD4B* | F | GATCACCGAGTTCTTGACCAC |
|  | R | TGGTTTCGCTCTCTACTTCGAT |
| Homo-*PLPP1* | F | GGCAGGTTGTCCTTCTATTCAG |
|  | R | CAGTGTGGGGCGTAAGAGT |
| Homo-*EIF3H* | F | CAGATGGAAATGATGCGGAGC |
|  | R | AGTATGTGGACTGATACCAGCC |
| Homo-*RBBP6* | F | GGCGCTCATTTTCCAGGTCTA |
|  | R | GAGCGTGAACGTGTTGAACC |
| Homo-*SRSF1* | F | ATGTCGGGAGGTGGTGTGATTC |
|  | R | TGTTCCACGGCCGCTTCGAG |
| Homo-*GALNT5* | F | TTGGAACATACGACCCTGGC |
|  | R | CACCACACATCCACACCTTG |
| Homo-*EREG* | F | GCTCCTTCATCGAATGCTAAAC |
|  | R | CCTAGCACATGCCAGGATAAA |
| Homo-*KLF5* | F | TCAGTCGTAGACCAGTTCTTCA |
|  | R | CTGGGATTTGTAGAGGCCAGT |
| Homo-*FENDRR* | F | CCACATGGATGGTTGCCACTCTC |
|  | R | GCTGGTACTCGGCCTTCTAATTGG |
| Homo-*TPT1-AS1* | F | GGGGTGAGCTCGTCGAAGTTCT |
|  | R | GGGCTGCTGCTGGCGCTGGT |
| Homo-*PXN-AS1* | F | AGAAGCTGCCGTTCACGAAT |
|  | R | ATGGGAAACGCCTAGCAAGTA |
| Homo-*U6* | F | CTCGCTTCGGCAGCACA |
|  | R | AACGCTTCACGAATTTGCGT |
| *hsa-miR-15b-5p*(RT) | Single strand | GTCGTATCCAGTGCAGGGTCCGAGGT  ATTCGCACTGGATACGACTGTAAA |
| *hsa-miR-15b-5p* | F | CGCGTAGCAGCACATCATGG |
|  | R | AGTGCAGGGTCCGAGGTATT |
| *hsa-miR-29c-3p*(RT) | Single strand | GTCGTATCCAGTGCAGGGTCCGAGGT  ATTCGCACTGGATACGACTAACCG |
| *hsa-miR-29c-3p* | F | CGCGTAGCACCATTTGAAAT |
|  | R | AGTGCAGGGTCCGAGGTATT |
| Homo-*FENDRR*-ASO | Single strand | TTTCAGTTCTGTCGTCGTTT |
| Homo-*TPT1-AS1*-ASO | Single strand | GAAGATGGGCAAGGATAGCC |
| Negative control-ASO | Single strand | GCGUATTATAGCCGAUUAAC |
| *hsa-miR-15b-5p* inhibitors | Single strand | UGUAAACCAUGAUGUGCUGCUA |
| *hsa-miR-15b-5p* inhibitors NC | Single strand | CAGUACUUUUGUGUAGUACAA |

**Table S3.** Primary antibodies used in this study.

| Antigens | Manufacturer | Catalog Number | Application |
| --- | --- | --- | --- |
| EIF3H | Santa Cruz | sc-271283 | 1:100 for IHC |
| RBBP6 | Santa Cruz | sc-9962 | 1:100 for IHC |
| SRSF1 | Santa Cruz | sc-33652 | 1:100 for IHC |

**Table S4.** The types, chromosome distributions, and subcellular localizations of DElncRNAs.

| DElncRNAs | Chromosome | Type | Subcellular localization |
| --- | --- | --- | --- |
| *HRNR* | 1 | sense_intronic | Cytoplasm |
| *Z97200.1* | 1 | sense_intronic | Cytoplasm |
| *TPT1-AS1* | 13 | antisense | Cytoplasm, Cytosol, Nucleus,  Ribosome, Exosome |
| *AC099791.2* | 1 | antisense | Exosome |
| *AL451042.1* | 1 | lincRNA | Cytoplasm |
| *AC138305.1* | 16 | lincRNA | Cytoplasm |
| *AL355488.1* | 1 | antisense | Cytoplasm, Cytosol |
| *AC078909.2* | 15 | lincRNA | Nucleus |
| *AP001107.3* | 11 | antisense | Cytoplasm |
| *AC107464.3* | 4 | antisense | Nucleus |
| *AL390719.2* | 1 | lincRNA | Ribosome |
| *PXN-AS1* | 12 | antisense | Cytoplasm, Cytosol, Nucleus,  Ribosome |
| *AL355102.1* | 14 | sense_intronic | Cytosol |
| *AC080038.2* | 17 | lincRNA | Cytoplasm |
| *AP000692.2* | 21 | antisense | Exosome |
| *LINC00210* | 1 | lincRNA | Nucleus |
| *AC022509.4* | 12 | antisense | Cytoplasm |
| *AC107375.1* | 8 | lincRNA | Ribosome |
| *AC009387.1* | 12 | lincRNA | Cytoplasm |
| *C18orf65* | 18 | lincRNA | Nucleus |
| *KIF25-AS1* | 6 | antisense | Cytosol, Nucleus, Ribosome |
| *AL138899.1* | 1 | lincRNA | Cytosol |
| *AC061992.2* | 17 | lincRNA | Cytoplasm |
| *LINC00922* | 16 | lincRNA | Cytoplasm |
| *LINC01068* | 13 | lincRNA | Cytoplasm |
| *AC010768.2* | 11 | antisense | Cytoplasm |
| *FENDRR* | 16 | lincRNA | Cytoplasm, Cytosol, Nucleus |
| *AC092316.1* | 19 | antisense | Nucleus |
| *LINC00682* | 4 | lincRNA | Cytoplasm, Cytosol, Ribosome,  Exosome |
| *AC099811.4* | 17 | antisense | Cytoplasm |

**Table S5.** The RNAs of the ceRNA network.

| Gene type | Gene symbol |
| --- | --- |
| lncRNA | *AL355488.1, FENDRR, LINC00922* |
| miRNA | *hsa-miR-29c-3p, hsa-miR-15b-5p* |
| mRNA | *PLPP1, MT1G, DACT2, CHGA, CAMKK2, HMGN3, ARL4A, TRAM2, SRSF1, EREG, FRMD4B, SAR1B, BMF, NCS1, CHMP4B, FBXO3, KLF5, RBBP6, TAB1, RNF222, GALNT5, ARFGAP2, CHAC1, EIF3H, LOX, KIAA0232, EMC2, PSMA4, KLF2, SH3RF3, DLL4* |

**Table S6.** Predicted binding affinity between SRSF1 and interacting lncRNAs using the catRAPID database.

|  | catRAPID^omics^ | |  | catRAPID^OmiXcore^ |
| --- | --- | --- | --- | --- |
|  | Interaction propensity | Z score |  | Interaction score |
| *TPT1-AS1* | 3.73 | -0.81 |  | 0.11 |
| *AL138899.1* | 3.25 | -1.07 |  | 0.57 |
| *PXN-AS1* | 2.6 | -0.7 |  | 0.20 |
| *AC107375.1* | 4.23 | -1.13 |  | 0.76 |
| *LINC00922* | 2.12 | -1.06 |  | 0.35 |
| *FENDRR* | 4.96 | -1.23 |  | 0.27 |
| *AL355488.1* | 4.62 | -1.22 |  | 0.07 |

**Table S7.** Spearman correlation coefficient calculation of RNAs interacted with SRSF1.

|  | *TPT1-AS1* | |  | *AL138899.1* | |  | *PXN-AS1* | |  | *AC107375.1* | |  | *LINC00922* | |  | *FENDRR* | |  | *AL355488.1* | |
| --- | --- | --- | --- | --- | --- | --- | --- | --- | --- | --- | --- | --- | --- | --- | --- | --- | --- | --- | --- | --- |
|  | rho | P-value |  | rho | P-value |  | rho | P-value |  | rho | P-value |  | rho | P-value |  | rho | P-value |  | rho | P-value |
| *ST7* | -0.6000 | 0.2080 |  | 0.8286 | 0.0416 |  | -0.6571 | 0.1562 |  | 0.9429 | 0.0048 |  | 0.6571 | 0.1562 |  | 0.7714 | 0.0724 |  | -0.9429 | 0.0048 |
| *FLT4* | 0.7714 | 0.0724 |  | -0.7714 | 0.0724 |  | 0.6000 | 0.2080 |  | -0.7714 | 0.0724 |  | -0.4286 | 0.3965 |  | -0.6000 | 0.2080 |  | 0.9429 | 0.0048 |
| *PSMA4* | -0.3143 | 0.5441 |  | 0.3143 | 0.5441 |  | -0.1429 | 0.7872 |  | 0.5429 | 0.2657 |  | 0.8857 | 0.0188 |  | 0.8286 | 0.0416 |  | -0.2571 | 0.6228 |
| *TRAM2* | 0.7143 | 0.1108 |  | -0.6000 | 0.2080 |  | 0.9429 | 0.0048 |  | -0.8286 | 0.0416 |  | -0.5429 | 0.2657 |  | -0.6571 | 0.1562 |  | 0.7143 | 0.1108 |
| *PLPP1* | -1.0000 | 0.0000 |  | 0.3143 | 0.5441 |  | -0.4857 | 0.3287 |  | 0.5429 | 0.2657 |  | 0.3714 | 0.4685 |  | 0.7143 | 0.1108 |  | -0.6571 | 0.1562 |
| *LIMS2* | 0.7714 | 0.0724 |  | -0.5429 | 0.2657 |  | 0.8857 | 0.0188 |  | -0.7714 | 0.0724 |  | -0.3714 | 0.4685 |  | -0.6000 | 0.2080 |  | 0.7714 | 0.0724 |
| *EDN1* | 0.8857 | 0.0188 |  | -0.3714 | 0.4685 |  | 0.7143 | 0.1108 |  | -0.6571 | 0.1562 |  | -0.3143 | 0.5441 |  | -0.6571 | 0.1562 |  | 0.7143 | 0.1108 |
| *LYZ* | 0.7714 | 0.0724 |  | -0.4857 | 0.3287 |  | 0.6571 | 0.1562 |  | -0.7714 | 0.0724 |  | -0.4857 | 0.3287 |  | -0.7714 | 0.0724 |  | 0.7714 | 0.0724 |
| *TAB1* | 0.7143 | 0.1108 |  | -0.6000 | 0.2080 |  | 0.9429 | 0.0048 |  | -0.8286 | 0.0416 |  | -0.5429 | 0.2657 |  | -0.6571 | 0.1562 |  | 0.7143 | 0.1108 |
| *CHMP4B* | -0.6571 | 0.1562 |  | 0.6000 | 0.2080 |  | -0.7714 | 0.0724 |  | 0.8857 | 0.0188 |  | 0.8286 | 0.0416 |  | 0.8857 | 0.0188 |  | -0.6571 | 0.1562 |
| *KLF5* | 0.4286 | 0.3965 |  | -0.7714 | 0.0724 |  | 0.2571 | 0.6228 |  | -0.7714 | 0.0724 |  | -0.9429 | 0.0048 |  | -0.8286 | 0.0416 |  | 0.6571 | 0.1562 |
| *BMF* | 0.7143 | 0.1108 |  | -0.8286 | 0.0416 |  | 0.6571 | 0.1562 |  | -0.8286 | 0.0416 |  | -0.6000 | 0.2080 |  | -0.6571 | 0.1562 |  | 0.8857 | 0.0188 |
| *EMC2* | -0.6571 | 0.1562 |  | 0.8286 | 0.0416 |  | -0.4857 | 0.3287 |  | 0.8857 | 0.0188 |  | 0.8857 | 0.0188 |  | 0.8857 | 0.0188 |  | -0.8286 | 0.0416 |
| *CLIP2* | 0.6000 | 0.2080 |  | -0.6000 | 0.2080 |  | 0.7714 | 0.0724 |  | -0.6000 | 0.2080 |  | -0.2571 | 0.6228 |  | -0.3143 | 0.5441 |  | 0.6571 | 0.1562 |
| *NCS1* | -0.6571 | 0.1562 |  | 0.8286 | 0.0416 |  | -0.4857 | 0.3287 |  | 0.8857 | 0.0188 |  | 0.8857 | 0.0188 |  | 0.8857 | 0.0188 |  | -0.8286 | 0.0416 |
| *FBXO3* | -0.6571 | 0.1562 |  | 0.6000 | 0.2080 |  | -0.7714 | 0.0724 |  | 0.8857 | 0.0188 |  | 0.8286 | 0.0416 |  | 0.8857 | 0.0188 |  | -0.6571 | 0.1562 |
| *CAMKK2* | 0.7143 | 0.1108 |  | -0.6000 | 0.2080 |  | 0.9429 | 0.0048 |  | -0.8286 | 0.0416 |  | -0.5429 | 0.2657 |  | -0.6571 | 0.1562 |  | 0.7143 | 0.1108 |
| *HBS1L* | -0.4857 | 0.3287 |  | 0.5429 | 0.2657 |  | -0.2000 | 0.7040 |  | 0.7143 | 0.1108 |  | 0.9429 | 0.0048 |  | 0.9429 | 0.0048 |  | -0.5429 | 0.2657 |
| *LOX* | 0.5429 | 0.2657 |  | -0.8857 | 0.0188 |  | 0.7143 | 0.1108 |  | -1.0000 | 0.0000 |  | -0.8286 | 0.0416 |  | -0.8286 | 0.0416 |  | 0.8857 | 0.0188 |
| *FRMD4B* | -0.7143 | 0.1108 |  | 0.6000 | 0.2080 |  | -0.9429 | 0.0048 |  | 0.8286 | 0.0416 |  | 0.5429 | 0.2657 |  | 0.6571 | 0.1562 |  | -0.7143 | 0.1108 |
| *HMGN3* | 0.8286 | 0.0416 |  | -0.5429 | 0.2657 |  | 0.7143 | 0.1108 |  | -0.7143 | 0.1108 |  | -0.6000 | 0.2080 |  | -0.7143 | 0.1108 |  | 0.6571 | 0.1562 |
| *NPC2* | -0.6571 | 0.1562 |  | 0.8286 | 0.0416 |  | -0.4857 | 0.3287 |  | 0.8857 | 0.0188 |  | 0.8857 | 0.0188 |  | 0.8857 | 0.0188 |  | -0.8286 | 0.0416 |
| *RBBP6* | 0.6000 | 0.2080 |  | -0.7143 | 0.1108 |  | 0.8857 | 0.0188 |  | -0.9429 | 0.0048 |  | -0.7143 | 0.1108 |  | -0.7714 | 0.0724 |  | 0.7714 | 0.0724 |
| *ARL4A* | -0.6571 | 0.1562 |  | 0.6000 | 0.2080 |  | -0.7714 | 0.0724 |  | 0.8857 | 0.0188 |  | 0.8286 | 0.0416 |  | 0.8857 | 0.0188 |  | -0.6571 | 0.1562 |
| *PI3* | 0.9429 | 0.0048 |  | -0.4857 | 0.3287 |  | 0.3143 | 0.5441 |  | -0.6000 | 0.2080 |  | -0.4857 | 0.3287 |  | -0.7714 | 0.0724 |  | 0.7714 | 0.0724 |
| *EREG* | 0.6000 | 0.2080 |  | -0.6000 | 0.2080 |  | 0.0857 | 0.8717 |  | -0.6000 | 0.2080 |  | -0.4286 | 0.3965 |  | -0.6571 | 0.1562 |  | 0.8286 | 0.0416 |
| *MT1G* | 0.7143 | 0.1108 |  | -0.8286 | 0.0416 |  | 0.6571 | 0.1562 |  | -0.8286 | 0.0416 |  | -0.6000 | 0.2080 |  | -0.6571 | 0.1562 |  | 0.8857 | 0.0188 |
| *LRFN3* | 0.6000 | 0.2080 |  | -0.9429 | 0.0048 |  | 0.6000 | 0.2080 |  | -0.9429 | 0.0048 |  | -0.7714 | 0.0724 |  | -0.7714 | 0.0724 |  | 0.9429 | 0.0048 |
| *BAIAP2L2* | 0.2571 | 0.6228 |  | -0.9429 | 0.0048 |  | 0.6000 | 0.2080 |  | -0.9429 | 0.0048 |  | -0.7714 | 0.0724 |  | -0.6571 | 0.1562 |  | 0.8286 | 0.0416 |
| *DLL4* | 0.3714 | 0.4685 |  | -0.6000 | 0.2080 |  | 0.9429 | 0.0048 |  | -0.8286 | 0.0416 |  | -0.5429 | 0.2657 |  | -0.5429 | 0.2657 |  | 0.6000 | 0.2080 |
| *CHAC1* | -0.3714 | 0.4685 |  | 0.7143 | 0.1108 |  | -0.3714 | 0.4685 |  | 0.8286 | 0.0416 |  | 1.0000 | 0.0000 |  | 0.8857 | 0.0188 |  | -0.6000 | 0.2080 |
| *SLC27A1* | 0.0857 | 0.8717 |  | 0.5429 | 0.2657 |  | -0.3714 | 0.4685 |  | 0.2571 | 0.6228 |  | 0.0286 | 0.9572 |  | -0.2000 | 0.7040 |  | -0.3143 | 0.5441 |
| *RNASEH2B* | 0.6000 | 0.2080 |  | -0.7143 | 0.1108 |  | 0.8857 | 0.0188 |  | -0.9429 | 0.0048 |  | -0.7143 | 0.1108 |  | -0.7714 | 0.0724 |  | 0.7714 | 0.0724 |
| *SRSF1* | 0.0286 | 0.9572 |  | -0.7714 | 0.0724 |  | 0.0857 | 0.8717 |  | -0.4857 | 0.3287 |  | -0.2000 | 0.7040 |  | -0.1429 | 0.7872 |  | 0.7143 | 0.1108 |
| *GALNT5* | 0.2571 | 0.6228 |  | -0.9429 | 0.0048 |  | 0.6000 | 0.2080 |  | -0.9429 | 0.0048 |  | -0.7714 | 0.0724 |  | -0.6571 | 0.1562 |  | 0.8286 | 0.0416 |
| *AQP10* | -0.6000 | 0.2080 |  | 0.7714 | 0.0724 |  | -0.6000 | 0.2080 |  | 0.9429 | 0.0048 |  | 0.9429 | 0.0048 |  | 0.9429 | 0.0048 |  | -0.7714 | 0.0724 |
| *EIF3H* | -0.7714 | 0.0724 |  | 0.4286 | 0.3965 |  | -0.6000 | 0.2080 |  | 0.7714 | 0.0724 |  | 0.7714 | 0.0724 |  | 0.9429 | 0.0048 |  | -0.6000 | 0.2080 |
| *ARFGAP2* | 0.6000 | 0.2080 |  | -0.9429 | 0.0048 |  | 0.6000 | 0.2080 |  | -0.9429 | 0.0048 |  | -0.7714 | 0.0724 |  | -0.7714 | 0.0724 |  | 0.9429 | 0.0048 |
| *SAR1B* | -0.8286 | 0.0416 |  | 0.2000 | 0.7040 |  | -0.3714 | 0.4685 |  | 0.3714 | 0.4685 |  | 0.4286 | 0.3965 |  | 0.6000 | 0.2080 |  | -0.3714 | 0.4685 |
| *HHEX* | -0.6571 | 0.1562 |  | 0.6000 | 0.2080 |  | -0.7714 | 0.0724 |  | 0.8857 | 0.0188 |  | 0.8286 | 0.0416 |  | 0.8857 | 0.0188 |  | -0.6571 | 0.1562 |
| *GRAP* | 0.7714 | 0.0724 |  | -0.7143 | 0.1108 |  | 0.3714 | 0.4685 |  | -0.7714 | 0.0724 |  | -0.5429 | 0.2657 |  | -0.7714 | 0.0724 |  | 0.9429 | 0.0048 |
| *MPZ* | 0.7714 | 0.0724 |  | -0.6571 | 0.1562 |  | 0.8286 | 0.0416 |  | -0.7714 | 0.0724 |  | -0.4857 | 0.3287 |  | -0.6000 | 0.2080 |  | 0.7714 | 0.0724 |
| *DCAF16* | 0.6571 | 0.1562 |  | -0.5429 | 0.2657 |  | 0.7143 | 0.1108 |  | -0.5429 | 0.2657 |  | -0.0857 | 0.8717 |  | -0.2571 | 0.6228 |  | 0.7143 | 0.1108 |
| *DACT2* | 0.6000 | 0.2080 |  | -0.7714 | 0.0724 |  | 0.6000 | 0.2080 |  | -0.9429 | 0.0048 |  | -0.9429 | 0.0048 |  | -0.9429 | 0.0048 |  | 0.7714 | 0.0724 |
| *GSDMA* | 0.6000 | 0.2080 |  | -0.9429 | 0.0048 |  | 0.6000 | 0.2080 |  | -0.9429 | 0.0048 |  | -0.7714 | 0.0724 |  | -0.7714 | 0.0724 |  | 0.9429 | 0.0048 |
| *TOR1AIP2* | -0.7143 | 0.1108 |  | 0.6000 | 0.2080 |  | -0.9429 | 0.0048 |  | 0.8286 | 0.0416 |  | 0.5429 | 0.2657 |  | 0.6571 | 0.1562 |  | -0.7143 | 0.1108 |
| *KIAA0232* | -0.3143 | 0.5441 |  | 0.8857 | 0.0188 |  | -0.5429 | 0.2657 |  | 0.8857 | 0.0188 |  | 0.6000 | 0.2080 |  | 0.6000 | 0.2080 |  | -0.8857 | 0.0188 |
| *C1GALT1C1* | -0.8286 | 0.0416 |  | 0.5429 | 0.2657 |  | -0.7143 | 0.1108 |  | 0.7143 | 0.1108 |  | 0.6000 | 0.2080 |  | 0.7143 | 0.1108 |  | -0.6571 | 0.1562 |
| *SH3RF3* | 0.5429 | 0.2657 |  | -0.7714 | 0.0724 |  | 0.6000 | 0.2080 |  | -0.6571 | 0.1562 |  | -0.3714 | 0.4685 |  | -0.3714 | 0.4685 |  | 0.7714 | 0.0724 |
| *ARV1* | -0.4286 | 0.3965 |  | 0.4857 | 0.3287 |  | -0.1429 | 0.7872 |  | 0.4286 | 0.3965 |  | 0.6571 | 0.1562 |  | 0.5429 | 0.2657 |  | -0.3714 | 0.4685 |
| *SAMD4B* | 0.7714 | 0.0724 |  | -0.7714 | 0.0724 |  | 0.6000 | 0.2080 |  | -0.7714 | 0.0724 |  | -0.4286 | 0.3965 |  | -0.6000 | 0.2080 |  | 0.9429 | 0.0048 |
| *ZBTB2* | -0.6000 | 0.2080 |  | 0.7714 | 0.0724 |  | -0.6000 | 0.2080 |  | 0.9429 | 0.0048 |  | 0.9429 | 0.0048 |  | 0.9429 | 0.0048 |  | -0.7714 | 0.0724 |
| *PMEL* | 0.8857 | 0.0188 |  | -0.3714 | 0.4685 |  | 0.7143 | 0.1108 |  | -0.6571 | 0.1562 |  | -0.3143 | 0.5441 |  | -0.6571 | 0.1562 |  | 0.7143 | 0.1108 |
| *RNF222* | -0.5798 | 0.2278 |  | 0.4928 | 0.3206 |  | -0.1449 | 0.7841 |  | 0.6377 | 0.1731 |  | 0.5508 | 0.2574 |  | 0.7827 | 0.0657 |  | -0.7247 | 0.1032 |
| *RPL37A* | -0.6000 | 0.2080 |  | 0.7714 | 0.0724 |  | -0.6000 | 0.2080 |  | 0.9429 | 0.0048 |  | 0.9429 | 0.0048 |  | 0.9429 | 0.0048 |  | -0.7714 | 0.0724 |
| *BRD3OS* | 0.6000 | 0.2080 |  | -0.6000 | 0.2080 |  | 0.7714 | 0.0724 |  | -0.6000 | 0.2080 |  | -0.2571 | 0.6228 |  | -0.3143 | 0.5441 |  | 0.6571 | 0.1562 |
| *AMY2A* | -0.4286 | 0.3965 |  | 0.7714 | 0.0724 |  | -0.2571 | 0.6228 |  | 0.7714 | 0.0724 |  | 0.9429 | 0.0048 |  | 0.8286 | 0.0416 |  | -0.6571 | 0.1562 |
| *SHANK3* | 0.7143 | 0.1108 |  | -0.8286 | 0.0416 |  | 0.6571 | 0.1562 |  | -0.8286 | 0.0416 |  | -0.6000 | 0.2080 |  | -0.6571 | 0.1562 |  | 0.8857 | 0.0188 |

**Table S8.** SRSF1-interacted DElncRNAs and their targets.

| LncRNA | Target | rho | P-value |
| --- | --- | --- | --- |
| *TPT1-AS1* | *PLPP1* | -1.0000 | 0.0000 |
|  | *PI3* | 0.9429 | 0.0048 |
| *AL138899.1* | *LRFN3* | -0.9429 | 0.0048 |
|  | *BAIAP2L2* | -0.9429 | 0.0048 |
|  | *GALNT5* | -0.9429 | 0.0048 |
|  | *ARFGAP2* | -0.9429 | 0.0048 |
|  | *GSDMA* | -0.9429 | 0.0048 |
| *PXN-AS1* | *TRAM2* | 0.9429 | 0.0048 |
|  | *TAB1* | 0.9429 | 0.0048 |
|  | *CAMKK2* | 0.9429 | 0.0048 |
|  | *FRMD4B* | -0.9429 | 0.0048 |
|  | *DLL4* | 0.9429 | 0.0048 |
|  | *TOR1AIP2* | -0.9429 | 0.0048 |
| *AC107375.1* | *ST7* | 0.9429 | 0.0048 |
|  | *LOX* | -1.0000 | 0.0000 |
|  | *RBBP6* | -0.9429 | 0.0048 |
|  | *LRFN3* | -0.9429 | 0.0048 |
|  | *BAIAP2L2* | -0.9429 | 0.0048 |
|  | *RNASEH2B* | -0.9429 | 0.0048 |
|  | *GALNT5* | -0.9429 | 0.0048 |
|  | *AQP10* | 0.9429 | 0.0048 |
|  | *ARFGAP2* | -0.9429 | 0.0048 |
|  | *DACT2* | -0.9429 | 0.0048 |
|  | *GSDMA* | -0.9429 | 0.0048 |
|  | *ZBTB2* | 0.9429 | 0.0048 |
|  | *RPL37A* | 0.9429 | 0.0048 |
| *LINC00922* | *KLF5* | -0.9429 | 0.0048 |
|  | *HBS1L* | 0.9429 | 0.0048 |
|  | *CHAC1* | 1.0000 | 0.0000 |
|  | *AQP10* | 0.9429 | 0.0048 |
|  | *DACT2* | -0.9429 | 0.0048 |
|  | *ZBTB2* | 0.9429 | 0.0048 |
|  | *RPL37A* | 0.9429 | 0.0048 |
|  | *AMY2A* | 0.9429 | 0.0048 |
| *FENDRR* | *HBS1L* | 0.9429 | 0.0048 |
|  | *AQP10* | 0.9429 | 0.0048 |
|  | *EIF3H* | 0.9429 | 0.0048 |
|  | *DACT2* | -0.9429 | 0.0048 |
|  | *ZBTB2* | 0.9429 | 0.0048 |
|  | *RPL37A* | 0.9429 | 0.0048 |
| *AL355488.1* | *ST7* | -0.9429 | 0.0048 |
|  | *FLT4* | 0.9429 | 0.0048 |
|  | *LRFN3* | 0.9429 | 0.0048 |
|  | *ARFGAP2* | 0.9429 | 0.0048 |
|  | *GRAP* | 0.9429 | 0.0048 |
|  | *GSDMA* | 0.9429 | 0.0048 |
|  | *SAMD4B* | 0.9429 | 0.0048 |

**Table S9.** Spearman correlation coefficient calculation of nucleus-localized DElncRNAs and DEmRNAs.

|  | *TPT1-AS1* | |  | *AC078909.2* | |  | *AC107464.3* | |  | *PXN-AS1* | |  | *LINC00210* | |  | *C18orf65* | |  | *KIF25-AS1* | |  | *FENDRR* | |  | *AC092316.1* | |
| --- | --- | --- | --- | --- | --- | --- | --- | --- | --- | --- | --- | --- | --- | --- | --- | --- | --- | --- | --- | --- | --- | --- | --- | --- | --- | --- |
|  | rho | P-value |  | rho | P-value |  | rho | P-value |  | rho | P-value |  | rho | P-value |  | rho | P-value |  | rho | P-value |  | rho | P-value |  | rho | P-value |
| *LCE3D* | 0.6000 | 0.2080 |  | 0.9429 | 0.0048 |  | 0.7714 | 0.0724 |  | 0.6571 | 0.1562 |  | -0.7714 | 0.0724 |  | -0.9429 | 0.0048 |  | -0.8857 | 0.0188 |  | -0.7714 | 0.0724 |  | -0.8286 | 0.0416 |
| *CCER2* | 0.6000 | 0.2080 |  | 0.9429 | 0.0048 |  | 0.7714 | 0.0724 |  | 0.6571 | 0.1562 |  | -0.7714 | 0.0724 |  | -0.9429 | 0.0048 |  | -0.8857 | 0.0188 |  | -0.7714 | 0.0724 |  | -0.8286 | 0.0416 |
| *LCE3E* | 0.6000 | 0.2080 |  | 0.9429 | 0.0048 |  | 0.7714 | 0.0724 |  | 0.6571 | 0.1562 |  | -0.7714 | 0.0724 |  | -0.9429 | 0.0048 |  | -0.8857 | 0.0188 |  | -0.7714 | 0.0724 |  | -0.8286 | 0.0416 |
| *MT1G* | 0.7143 | 0.1108 |  | 0.8857 | 0.0188 |  | 0.8286 | 0.0416 |  | 0.6571 | 0.1562 |  | -0.6571 | 0.1562 |  | -0.6571 | 0.1562 |  | -0.9429 | 0.0048 |  | -0.6571 | 0.1562 |  | -0.7714 | 0.0724 |
| *SHANK3* | 0.7143 | 0.1108 |  | 0.8857 | 0.0188 |  | 0.8286 | 0.0416 |  | 0.6571 | 0.1562 |  | -0.6571 | 0.1562 |  | -0.6571 | 0.1562 |  | -0.9429 | 0.0048 |  | -0.6571 | 0.1562 |  | -0.7714 | 0.0724 |
| *FLT4* | 0.7714 | 0.0724 |  | 0.9429 | 0.0048 |  | 0.7714 | 0.0724 |  | 0.6000 | 0.2080 |  | -0.6000 | 0.2080 |  | -0.7714 | 0.0724 |  | -0.8857 | 0.0188 |  | -0.6000 | 0.2080 |  | -0.6571 | 0.1562 |
| *MT1HL1* | 0.6000 | 0.2080 |  | 0.9429 | 0.0048 |  | 0.7714 | 0.0724 |  | 0.6571 | 0.1562 |  | -0.7714 | 0.0724 |  | -0.9429 | 0.0048 |  | -0.8857 | 0.0188 |  | -0.7714 | 0.0724 |  | -0.8286 | 0.0416 |
| *GRAP* | 0.7714 | 0.0724 |  | 0.9429 | 0.0048 |  | 0.6000 | 0.2080 |  | 0.3714 | 0.4685 |  | -0.7714 | 0.0724 |  | -0.9429 | 0.0048 |  | -0.8286 | 0.0416 |  | -0.7714 | 0.0724 |  | -0.7143 | 0.1108 |
| *DACT2* | 0.6000 | 0.2080 |  | 0.7714 | 0.0724 |  | 0.7714 | 0.0724 |  | 0.6000 | 0.2080 |  | -0.9429 | 0.0048 |  | -0.7714 | 0.0724 |  | -0.8857 | 0.0188 |  | -0.9429 | 0.0048 |  | -1.0000 | 0.0000 |
| *PI3* | 0.9429 | 0.0048 |  | 0.7714 | 0.0724 |  | 0.6000 | 0.2080 |  | 0.3143 | 0.5441 |  | -0.7714 | 0.0724 |  | -0.7714 | 0.0724 |  | -0.7143 | 0.1108 |  | -0.7714 | 0.0724 |  | -0.6571 | 0.1562 |
| *CHGA* | 0.6000 | 0.2080 |  | 0.9429 | 0.0048 |  | 0.7714 | 0.0724 |  | 0.6571 | 0.1562 |  | -0.7714 | 0.0724 |  | -0.9429 | 0.0048 |  | -0.8857 | 0.0188 |  | -0.7714 | 0.0724 |  | -0.8286 | 0.0416 |
| *EDN1* | 0.8857 | 0.0188 |  | 0.7143 | 0.1108 |  | 0.8286 | 0.0416 |  | 0.7143 | 0.1108 |  | -0.6571 | 0.1562 |  | -0.8286 | 0.0416 |  | -0.6000 | 0.2080 |  | -0.6571 | 0.1562 |  | -0.6000 | 0.2080 |
| *CAMKK2* | 0.7143 | 0.1108 |  | 0.7143 | 0.1108 |  | 1.0000 | 0.0000 |  | 0.9429 | 0.0048 |  | -0.6571 | 0.1562 |  | -0.6571 | 0.1562 |  | -0.7714 | 0.0724 |  | -0.6571 | 0.1562 |  | -0.7714 | 0.0724 |
| *LRFN3* | 0.6000 | 0.2080 |  | 0.9429 | 0.0048 |  | 0.7714 | 0.0724 |  | 0.6000 | 0.2080 |  | -0.7714 | 0.0724 |  | -0.7714 | 0.0724 |  | -1.0000 | 0.0000 |  | -0.7714 | 0.0724 |  | -0.8857 | 0.0188 |
| *HMGN3* | 0.8286 | 0.0416 |  | 0.6571 | 0.1562 |  | 0.8857 | 0.0188 |  | 0.7143 | 0.1108 |  | -0.7143 | 0.1108 |  | -0.5429 | 0.2657 |  | -0.7714 | 0.0724 |  | -0.7143 | 0.1108 |  | -0.7714 | 0.0724 |
| *SOX10* | 0.7714 | 0.0724 |  | 0.6000 | 0.2080 |  | 0.9429 | 0.0048 |  | 0.8286 | 0.0416 |  | -0.7714 | 0.0724 |  | -0.6000 | 0.2080 |  | -0.7143 | 0.1108 |  | -0.7714 | 0.0724 |  | -0.8286 | 0.0416 |
| *LCE3C* | 0.5429 | 0.2657 |  | 0.8857 | 0.0188 |  | 0.8286 | 0.0416 |  | 0.7143 | 0.1108 |  | -0.8286 | 0.0416 |  | -0.8286 | 0.0416 |  | -0.9429 | 0.0048 |  | -0.8286 | 0.0416 |  | -0.9429 | 0.0048 |
| *SAMD4B* | 0.7714 | 0.0724 |  | 0.9429 | 0.0048 |  | 0.7714 | 0.0724 |  | 0.6000 | 0.2080 |  | -0.6000 | 0.2080 |  | -0.7714 | 0.0724 |  | -0.8857 | 0.0188 |  | -0.6000 | 0.2080 |  | -0.6571 | 0.1562 |
| *PMEL* | 0.8857 | 0.0188 |  | 0.7143 | 0.1108 |  | 0.8286 | 0.0416 |  | 0.7143 | 0.1108 |  | -0.6571 | 0.1562 |  | -0.8286 | 0.0416 |  | -0.6000 | 0.2080 |  | -0.6571 | 0.1562 |  | -0.6000 | 0.2080 |
| *LIMS2* | 0.7714 | 0.0724 |  | 0.7714 | 0.0724 |  | 0.9429 | 0.0048 |  | 0.8857 | 0.0188 |  | -0.6000 | 0.2080 |  | -0.7714 | 0.0724 |  | -0.7143 | 0.1108 |  | -0.6000 | 0.2080 |  | -0.6571 | 0.1562 |
| *GSDMA* | 0.6000 | 0.2080 |  | 0.9429 | 0.0048 |  | 0.7714 | 0.0724 |  | 0.6000 | 0.2080 |  | -0.7714 | 0.0724 |  | -0.7714 | 0.0724 |  | -1.0000 | 0.0000 |  | -0.7714 | 0.0724 |  | -0.8857 | 0.0188 |
| *RNASEH2B* | 0.6000 | 0.2080 |  | 0.7714 | 0.0724 |  | 0.9429 | 0.0048 |  | 0.8857 | 0.0188 |  | -0.7714 | 0.0724 |  | -0.7714 | 0.0724 |  | -0.8286 | 0.0416 |  | -0.7714 | 0.0724 |  | -0.8857 | 0.0188 |
| *TRAM2* | 0.7143 | 0.1108 |  | 0.7143 | 0.1108 |  | 1.0000 | 0.0000 |  | 0.9429 | 0.0048 |  | -0.6571 | 0.1562 |  | -0.6571 | 0.1562 |  | -0.7714 | 0.0724 |  | -0.6571 | 0.1562 |  | -0.7714 | 0.0724 |
| *LYZ* | 0.7714 | 0.0724 |  | 0.7714 | 0.0724 |  | 0.7714 | 0.0724 |  | 0.6571 | 0.1562 |  | -0.7714 | 0.0724 |  | -0.9429 | 0.0048 |  | -0.6571 | 0.1562 |  | -0.7714 | 0.0724 |  | -0.7143 | 0.1108 |
| *SRSF1* | 0.0286 | 0.9572 |  | 0.7143 | 0.1108 |  | 0.1429 | 0.7872 |  | 0.0857 | 0.8717 |  | -0.1429 | 0.7872 |  | -0.4857 | 0.3287 |  | -0.6000 | 0.2080 |  | -0.1429 | 0.7872 |  | -0.2571 | 0.6228 |
| *EREG* | 0.6000 | 0.2080 |  | 0.8286 | 0.0416 |  | 0.3143 | 0.5441 |  | 0.0857 | 0.8717 |  | -0.6571 | 0.1562 |  | -0.8857 | 0.0188 |  | -0.6571 | 0.1562 |  | -0.6571 | 0.1562 |  | -0.5429 | 0.2657 |
| *SCNN1G* | -0.4286 | 0.3965 |  | -0.1429 | 0.7872 |  | 0.2000 | 0.7040 |  | 0.4286 | 0.3965 |  | -0.0286 | 0.9572 |  | 0.0286 | 0.9572 |  | 0.0286 | 0.9572 |  | -0.0286 | 0.9572 |  | -0.2000 | 0.7040 |
| *BMF* | 0.7143 | 0.1108 |  | 0.8857 | 0.0188 |  | 0.8286 | 0.0416 |  | 0.6571 | 0.1562 |  | -0.6571 | 0.1562 |  | -0.6571 | 0.1562 |  | -0.9429 | 0.0048 |  | -0.6571 | 0.1562 |  | -0.7714 | 0.0724 |
| *DCAF16* | 0.6571 | 0.1562 |  | 0.7143 | 0.1108 |  | 0.7714 | 0.0724 |  | 0.7143 | 0.1108 |  | -0.2571 | 0.6228 |  | -0.4857 | 0.3287 |  | -0.6571 | 0.1562 |  | -0.2571 | 0.6228 |  | -0.3714 | 0.4685 |
| *KLF5* | 0.4286 | 0.3965 |  | 0.6571 | 0.1562 |  | 0.4857 | 0.3287 |  | 0.2571 | 0.6228 |  | -0.8286 | 0.0416 |  | -0.5429 | 0.2657 |  | -0.8286 | 0.0416 |  | -0.8286 | 0.0416 |  | -0.8857 | 0.0188 |
| *RBBP6* | 0.6000 | 0.2080 |  | 0.7714 | 0.0724 |  | 0.9429 | 0.0048 |  | 0.8857 | 0.0188 |  | -0.7714 | 0.0724 |  | -0.7714 | 0.0724 |  | -0.8286 | 0.0416 |  | -0.7714 | 0.0724 |  | -0.8857 | 0.0188 |
| *TAB1* | 0.7143 | 0.1108 |  | 0.7143 | 0.1108 |  | 1.0000 | 0.0000 |  | 0.9429 | 0.0048 |  | -0.6571 | 0.1562 |  | -0.6571 | 0.1562 |  | -0.7714 | 0.0724 |  | -0.6571 | 0.1562 |  | -0.7714 | 0.0724 |
| *SPRR2G* | 0.5429 | 0.2657 |  | 0.6000 | 0.2080 |  | 0.8857 | 0.0188 |  | 0.9429 | 0.0048 |  | -0.3714 | 0.4685 |  | -0.6000 | 0.2080 |  | -0.5429 | 0.2657 |  | -0.3714 | 0.4685 |  | -0.4857 | 0.3287 |
| *GALNT5* | 0.2571 | 0.6228 |  | 0.8286 | 0.0416 |  | 0.6571 | 0.1562 |  | 0.6000 | 0.2080 |  | -0.6571 | 0.1562 |  | -0.7143 | 0.1108 |  | -0.8857 | 0.0188 |  | -0.6571 | 0.1562 |  | -0.8286 | 0.0416 |
| *SLC27A1* | 0.0857 | 0.8717 |  | -0.3143 | 0.5441 |  | -0.3143 | 0.5441 |  | -0.3714 | 0.4685 |  | -0.2000 | 0.7040 |  | -0.1429 | 0.7872 |  | 0.4286 | 0.3965 |  | -0.2000 | 0.7040 |  | 0.0857 | 0.8717 |
| *MPZ* | 0.7714 | 0.0724 |  | 0.7714 | 0.0724 |  | 0.9429 | 0.0048 |  | 0.8286 | 0.0416 |  | -0.6000 | 0.2080 |  | -0.6000 | 0.2080 |  | -0.8286 | 0.0416 |  | -0.6000 | 0.2080 |  | -0.7143 | 0.1108 |
| *ARFGAP2* | 0.6000 | 0.2080 |  | 0.9429 | 0.0048 |  | 0.7714 | 0.0724 |  | 0.6000 | 0.2080 |  | -0.7714 | 0.0724 |  | -0.7714 | 0.0724 |  | -1.0000 | 0.0000 |  | -0.7714 | 0.0724 |  | -0.8857 | 0.0188 |
| *CLIP2* | 0.6000 | 0.2080 |  | 0.6571 | 0.1562 |  | 0.8286 | 0.0416 |  | 0.7714 | 0.0724 |  | -0.3143 | 0.5441 |  | -0.3714 | 0.4685 |  | -0.7143 | 0.1108 |  | -0.3143 | 0.5441 |  | -0.4857 | 0.3287 |
| *LOX* | 0.5429 | 0.2657 |  | 0.8857 | 0.0188 |  | 0.8286 | 0.0416 |  | 0.7143 | 0.1108 |  | -0.8286 | 0.0416 |  | -0.8286 | 0.0416 |  | -0.9429 | 0.0048 |  | -0.8286 | 0.0416 |  | -0.9429 | 0.0048 |
| *SFRP1* | 0.3714 | 0.4685 |  | 0.6000 | 0.2080 |  | 0.8857 | 0.0188 |  | 0.9429 | 0.0048 |  | -0.5429 | 0.2657 |  | -0.6000 | 0.2080 |  | -0.6571 | 0.1562 |  | -0.5429 | 0.2657 |  | -0.7143 | 0.1108 |
| *KLF2* | 0.4286 | 0.3965 |  | 0.6571 | 0.1562 |  | 0.8286 | 0.0416 |  | 0.8857 | 0.0188 |  | -0.4857 | 0.3287 |  | -0.7143 | 0.1108 |  | -0.6000 | 0.2080 |  | -0.4857 | 0.3287 |  | -0.6000 | 0.2080 |
| *SH3RF3* | 0.5429 | 0.2657 |  | 0.7714 | 0.0724 |  | 0.7143 | 0.1108 |  | 0.6000 | 0.2080 |  | -0.3714 | 0.4685 |  | -0.4286 | 0.3965 |  | -0.8286 | 0.0416 |  | -0.3714 | 0.4685 |  | -0.5429 | 0.2657 |
| *DLL4* | 0.3714 | 0.4685 |  | 0.6000 | 0.2080 |  | 0.8857 | 0.0188 |  | 0.9429 | 0.0048 |  | -0.5429 | 0.2657 |  | -0.6000 | 0.2080 |  | -0.6571 | 0.1562 |  | -0.5429 | 0.2657 |  | -0.7143 | 0.1108 |
| *BAIAP2L2* | 0.2571 | 0.6228 |  | 0.8286 | 0.0416 |  | 0.6571 | 0.1562 |  | 0.6000 | 0.2080 |  | -0.6571 | 0.1562 |  | -0.7143 | 0.1108 |  | -0.8857 | 0.0188 |  | -0.6571 | 0.1562 |  | -0.8286 | 0.0416 |
| *BRD3OS* | 0.6000 | 0.2080 |  | 0.6571 | 0.1562 |  | 0.8286 | 0.0416 |  | 0.7714 | 0.0724 |  | -0.3143 | 0.5441 |  | -0.3714 | 0.4685 |  | -0.7143 | 0.1108 |  | -0.3143 | 0.5441 |  | -0.4857 | 0.3287 |
| *PLPP1* | -1.0000 | 0.0000 |  | -0.6571 | 0.1562 |  | -0.7143 | 0.1108 |  | -0.4857 | 0.3287 |  | 0.7143 | 0.1108 |  | 0.7143 | 0.1108 |  | 0.6000 | 0.2080 |  | 0.7143 | 0.1108 |  | 0.6000 | 0.2080 |
| *CPNE6* | -0.7714 | 0.0724 |  | -0.6000 | 0.2080 |  | -0.7714 | 0.0724 |  | -0.6000 | 0.2080 |  | 0.9429 | 0.0048 |  | 0.7714 | 0.0724 |  | 0.6571 | 0.1562 |  | 0.9429 | 0.0048 |  | 0.8857 | 0.0188 |
| *TOR1AIP2* | -0.7143 | 0.1108 |  | -0.7143 | 0.1108 |  | -1.0000 | 0.0000 |  | -0.9429 | 0.0048 |  | 0.6571 | 0.1562 |  | 0.6571 | 0.1562 |  | 0.7714 | 0.0724 |  | 0.6571 | 0.1562 |  | 0.7714 | 0.0724 |
| *C8orf48* | -0.7714 | 0.0724 |  | -0.7714 | 0.0724 |  | -0.9429 | 0.0048 |  | -0.8857 | 0.0188 |  | 0.6000 | 0.2080 |  | 0.7714 | 0.0724 |  | 0.7143 | 0.1108 |  | 0.6000 | 0.2080 |  | 0.6571 | 0.1562 |
| *RPL37A* | -0.6000 | 0.2080 |  | -0.7714 | 0.0724 |  | -0.7714 | 0.0724 |  | -0.6000 | 0.2080 |  | 0.9429 | 0.0048 |  | 0.7714 | 0.0724 |  | 0.8857 | 0.0188 |  | 0.9429 | 0.0048 |  | 1.0000 | 0.0000 |
| *ZBTB2* | -0.6000 | 0.2080 |  | -0.7714 | 0.0724 |  | -0.7714 | 0.0724 |  | -0.6000 | 0.2080 |  | 0.9429 | 0.0048 |  | 0.7714 | 0.0724 |  | 0.8857 | 0.0188 |  | 0.9429 | 0.0048 |  | 1.0000 | 0.0000 |
| *ARL4A* | -0.6571 | 0.1562 |  | -0.6571 | 0.1562 |  | -0.8857 | 0.0188 |  | -0.7714 | 0.0724 |  | 0.8857 | 0.0188 |  | 0.7143 | 0.1108 |  | 0.7714 | 0.0724 |  | 0.8857 | 0.0188 |  | 0.9429 | 0.0048 |
| *FRMD4B* | -0.7143 | 0.1108 |  | -0.7143 | 0.1108 |  | -1.0000 | 0.0000 |  | -0.9429 | 0.0048 |  | 0.6571 | 0.1562 |  | 0.6571 | 0.1562 |  | 0.7714 | 0.0724 |  | 0.6571 | 0.1562 |  | 0.7714 | 0.0724 |
| *SAR1B* | -0.8286 | 0.0416 |  | -0.3714 | 0.4685 |  | -0.6000 | 0.2080 |  | -0.3714 | 0.4685 |  | 0.6000 | 0.2080 |  | 0.3143 | 0.5441 |  | 0.4857 | 0.3287 |  | 0.6000 | 0.2080 |  | 0.5429 | 0.2657 |
| *GFRA2* | -0.6000 | 0.2080 |  | -0.7714 | 0.0724 |  | -0.7714 | 0.0724 |  | -0.6000 | 0.2080 |  | 0.9429 | 0.0048 |  | 0.7714 | 0.0724 |  | 0.8857 | 0.0188 |  | 0.9429 | 0.0048 |  | 1.0000 | 0.0000 |
| *AQP10* | -0.6000 | 0.2080 |  | -0.7714 | 0.0724 |  | -0.7714 | 0.0724 |  | -0.6000 | 0.2080 |  | 0.9429 | 0.0048 |  | 0.7714 | 0.0724 |  | 0.8857 | 0.0188 |  | 0.9429 | 0.0048 |  | 1.0000 | 0.0000 |
| *HHEX* | -0.6571 | 0.1562 |  | -0.6571 | 0.1562 |  | -0.8857 | 0.0188 |  | -0.7714 | 0.0724 |  | 0.8857 | 0.0188 |  | 0.7143 | 0.1108 |  | 0.7714 | 0.0724 |  | 0.8857 | 0.0188 |  | 0.9429 | 0.0048 |
| *NCS1* | -0.6571 | 0.1562 |  | -0.8286 | 0.0416 |  | -0.7143 | 0.1108 |  | -0.4857 | 0.3287 |  | 0.8857 | 0.0188 |  | 0.7143 | 0.1108 |  | 0.9429 | 0.0048 |  | 0.8857 | 0.0188 |  | 0.9429 | 0.0048 |
| *CHMP4B* | -0.6571 | 0.1562 |  | -0.6571 | 0.1562 |  | -0.8857 | 0.0188 |  | -0.7714 | 0.0724 |  | 0.8857 | 0.0188 |  | 0.7143 | 0.1108 |  | 0.7714 | 0.0724 |  | 0.8857 | 0.0188 |  | 0.9429 | 0.0048 |
| *FBXO3* | -0.6571 | 0.1562 |  | -0.6571 | 0.1562 |  | -0.8857 | 0.0188 |  | -0.7714 | 0.0724 |  | 0.8857 | 0.0188 |  | 0.7143 | 0.1108 |  | 0.7714 | 0.0724 |  | 0.8857 | 0.0188 |  | 0.9429 | 0.0048 |
| *HBS1L* | -0.4857 | 0.3287 |  | -0.5429 | 0.2657 |  | -0.4286 | 0.3965 |  | -0.2000 | 0.7040 |  | 0.9429 | 0.0048 |  | 0.6571 | 0.1562 |  | 0.6571 | 0.1562 |  | 0.9429 | 0.0048 |  | 0.8857 | 0.0188 |
| *RNF222* | -0.5798 | 0.2278 |  | -0.7247 | 0.1032 |  | -0.3479 | 0.4993 |  | -0.1449 | 0.7841 |  | 0.7827 | 0.0657 |  | 0.9276 | 0.0077 |  | 0.5798 | 0.2278 |  | 0.7827 | 0.0657 |  | 0.6377 | 0.1731 |
| *AMY2A* | -0.4286 | 0.3965 |  | -0.6571 | 0.1562 |  | -0.4857 | 0.3287 |  | -0.2571 | 0.6228 |  | 0.8286 | 0.0416 |  | 0.5429 | 0.2657 |  | 0.8286 | 0.0416 |  | 0.8286 | 0.0416 |  | 0.8857 | 0.0188 |
| *CHAC1* | -0.3714 | 0.4685 |  | -0.6000 | 0.2080 |  | -0.5429 | 0.2657 |  | -0.3714 | 0.4685 |  | 0.8857 | 0.0188 |  | 0.6000 | 0.2080 |  | 0.7714 | 0.0724 |  | 0.8857 | 0.0188 |  | 0.9429 | 0.0048 |
| *C1GALT1C1* | -0.8286 | 0.0416 |  | -0.6571 | 0.1562 |  | -0.8857 | 0.0188 |  | -0.7143 | 0.1108 |  | 0.7143 | 0.1108 |  | 0.5429 | 0.2657 |  | 0.7714 | 0.0724 |  | 0.7143 | 0.1108 |  | 0.7714 | 0.0724 |
| *GRHL2* | -0.5429 | 0.2657 |  | -0.8857 | 0.0188 |  | -0.8286 | 0.0416 |  | -0.7143 | 0.1108 |  | 0.8286 | 0.0416 |  | 0.8286 | 0.0416 |  | 0.9429 | 0.0048 |  | 0.8286 | 0.0416 |  | 0.9429 | 0.0048 |
| *EIF3H* | -0.7714 | 0.0724 |  | -0.6000 | 0.2080 |  | -0.7714 | 0.0724 |  | -0.6000 | 0.2080 |  | 0.9429 | 0.0048 |  | 0.7714 | 0.0724 |  | 0.6571 | 0.1562 |  | 0.9429 | 0.0048 |  | 0.8857 | 0.0188 |
| *ST7* | -0.6000 | 0.2080 |  | -0.9429 | 0.0048 |  | -0.7714 | 0.0724 |  | -0.6571 | 0.1562 |  | 0.7714 | 0.0724 |  | 0.9429 | 0.0048 |  | 0.8857 | 0.0188 |  | 0.7714 | 0.0724 |  | 0.8286 | 0.0416 |
| *KIAA0232* | -0.3143 | 0.5441 |  | -0.8857 | 0.0188 |  | -0.6000 | 0.2080 |  | -0.5429 | 0.2657 |  | 0.6000 | 0.2080 |  | 0.8286 | 0.0416 |  | 0.8286 | 0.0416 |  | 0.6000 | 0.2080 |  | 0.7143 | 0.1108 |
| *EMC2* | -0.6571 | 0.1562 |  | -0.8286 | 0.0416 |  | -0.7143 | 0.1108 |  | -0.4857 | 0.3287 |  | 0.8857 | 0.0188 |  | 0.7143 | 0.1108 |  | 0.9429 | 0.0048 |  | 0.8857 | 0.0188 |  | 0.9429 | 0.0048 |
| *PSMA4* | -0.3143 | 0.5441 |  | -0.2571 | 0.6228 |  | -0.3143 | 0.5441 |  | -0.1429 | 0.7872 |  | 0.8286 | 0.0416 |  | 0.4286 | 0.3965 |  | 0.4286 | 0.3965 |  | 0.8286 | 0.0416 |  | 0.7714 | 0.0724 |
| *NPC2* | -0.6571 | 0.1562 |  | -0.8286 | 0.0416 |  | -0.7143 | 0.1108 |  | -0.4857 | 0.3287 |  | 0.8857 | 0.0188 |  | 0.7143 | 0.1108 |  | 0.9429 | 0.0048 |  | 0.8857 | 0.0188 |  | 0.9429 | 0.0048 |
| *ARV1* | -0.4286 | 0.3965 |  | -0.3714 | 0.4685 |  | -0.3714 | 0.4685 |  | -0.1429 | 0.7872 |  | 0.5429 | 0.2657 |  | 0.1429 | 0.7872 |  | 0.6000 | 0.2080 |  | 0.5429 | 0.2657 |  | 0.6000 | 0.2080 |

**Table S10.** Nucleus-localized DElncRNAs and their candidate cis-targets.

| LncRNA | Target | rho | P-value |
| --- | --- | --- | --- |
| *AC078909.2*  （chr15:37,100,742-37,117,666） | *BMF*  （chr15:40,087,890-40,108,892） | 0.8857 | 0.0188 |
| *PXN-AS1*  （chr12:120,194,917-120,215,029） | *CAMKK2*  （chr12:121,237,675-121,298,308） | 0.9429 | 0.0048 |
| *LINC00210*  （chr1:217,892,899-217,920,805） | *LCE3C*  （chr1:152,600,234-152,601,086） | -0.8286 | 0.0416 |
|  | *AQP10*  （chr1:154,321,090-154,325,325） | 0.9429 | 0.0048 |
|  | *AMY2A*  （chr1:103,616,651-103,625,780） | 0.8286 | 0.0416 |
| *KIF25-AS1*  （chr6:167,975,947-167,997,186） | *DACT2*  （chr6:168,292,830-168,319,777） | -0.8857 | 0.0188 |
|  | *ZBTB2*  （chr6:151,364,115-151,391,559） | 0.8857 | 0.0188 |
| *AC092316.1*  （chr19:8,526,463-8,555,992） | *CCER2*  （chr19:38,908,980-38,912,186） | -0.8286 | 0.0416 |
|  | *LRFN3*  （chr19:35,935,358-35,946,624） | -0.8857 | 0.0188 |

**Table S11.** Nucleus-localized DElncRNAs and their trans-targets.

| LncRNA | Target | rho | P-value |
| --- | --- | --- | --- |
| *TPT1-AS1* | *PI3* | 0.9429 | 0.0048 |
|  | *PLPP1* | -1.0000 | 0.0000 |
| *AC078909.2* | *LCE3D* | 0.9429 | 0.0048 |
|  | *CCER2* | 0.9429 | 0.0048 |
|  | *LCE3E* | 0.9429 | 0.0048 |
|  | *FLT4* | 0.9429 | 0.0048 |
|  | *MT1HL1* | 0.9429 | 0.0048 |
|  | *GRAP* | 0.9429 | 0.0048 |
|  | *CHGA* | 0.9429 | 0.0048 |
|  | *LRFN3* | 0.9429 | 0.0048 |
|  | *SAMD4B* | 0.9429 | 0.0048 |
|  | *GSDMA* | 0.9429 | 0.0048 |
|  | *ARFGAP2* | 0.9429 | 0.0048 |
|  | *ST7* | -0.9429 | 0.0048 |
| *AC107464.3* | *CAMKK2* | 1.0000 | 0.0000 |
|  | *SOX10* | 0.9429 | 0.0048 |
|  | *LIMS2* | 0.9429 | 0.0048 |
|  | *RNASEH2B* | 0.9429 | 0.0048 |
|  | *TRAM2* | 1.0000 | 0.0000 |
|  | *RBBP6* | 0.9429 | 0.0048 |
|  | *TAB1* | 1.0000 | 0.0000 |
|  | *MPZ* | 0.9429 | 0.0048 |
|  | *TOR1AIP2* | -1.0000 | 0.0000 |
|  | *C8orf48* | -0.9429 | 0.0048 |
|  | *FRMD4B* | -1.0000 | 0.0000 |
| *PXN-AS1* | *CAMKK2* | 0.9429 | 0.0048 |
|  | *TRAM2* | 0.9429 | 0.0048 |
|  | *TAB1* | 0.9429 | 0.0048 |
|  | *SPRR2G* | 0.9429 | 0.0048 |
|  | *SFRP1* | 0.9429 | 0.0048 |
|  | *DLL4* | 0.9429 | 0.0048 |
|  | *TOR1AIP2* | -0.9429 | 0.0048 |
|  | *FRMD4B* | -0.9429 | 0.0048 |
| *LINC00210* | *DACT2* | -0.9429 | 0.0048 |
|  | *CPNE6* | 0.9429 | 0.0048 |
|  | *RPL37A* | 0.9429 | 0.0048 |
|  | *ZBTB2* | 0.9429 | 0.0048 |
|  | *GFRA2* | 0.9429 | 0.0048 |
|  | *AQP10* | 0.9429 | 0.0048 |
|  | *HBS1L* | 0.9429 | 0.0048 |
|  | *EIF3H* | 0.9429 | 0.0048 |
| *C18orf65* | *LCE3D* | -0.9429 | 0.0048 |
|  | *CCER2* | -0.9429 | 0.0048 |
|  | *LCE3E* | -0.9429 | 0.0048 |
|  | *MT1HL1* | -0.9429 | 0.0048 |
|  | *GRAP* | -0.9429 | 0.0048 |
|  | *CHGA* | -0.9429 | 0.0048 |
|  | *LYZ* | -0.9429 | 0.0048 |
|  | *RNF222* | 0.9276 | 0.0077 |
|  | *ST7* | 0.9429 | 0.0048 |
| *KIF25-AS1* | *MT1G* | -0.9429 | 0.0048 |
|  | *SHANK3* | -0.9429 | 0.0048 |
|  | *LRFN3* | -1.0000 | 0.0000 |
|  | *LCE3C* | -0.9429 | 0.0048 |
|  | *GSDMA* | -1.0000 | 0.0000 |
|  | *BMF* | -0.9429 | 0.0048 |
|  | *ARFGAP2* | -1.0000 | 0.0000 |
|  | *LOX* | -0.9429 | 0.0048 |
|  | *NCS1* | 0.9429 | 0.0048 |
|  | *GRHL2* | 0.9429 | 0.0048 |
|  | *EMC2* | 0.9429 | 0.0048 |
|  | *NPC2* | 0.9429 | 0.0048 |
| *FENDRR* | *DACT2* | -0.9429 | 0.0048 |
|  | *CPNE6* | 0.9429 | 0.0048 |
|  | *RPL37A* | 0.9429 | 0.0048 |
|  | *ZBTB2* | 0.9429 | 0.0048 |
|  | *GFRA2* | 0.9429 | 0.0048 |
|  | *AQP10* | 0.9429 | 0.0048 |
|  | *HBS1L* | 0.9429 | 0.0048 |
|  | *EIF3H* | 0.9429 | 0.0048 |
| *AC092316.1* | *DACT2* | -1.0000 | 0.0000 |
|  | *LCE3C* | -0.9429 | 0.0048 |
|  | *LOX* | -0.9429 | 0.0048 |
|  | *RPL37A* | 1.0000 | 0.0000 |
|  | *ZBTB2* | 1.0000 | 0.0000 |
|  | *ARL4A* | 0.9429 | 0.0048 |
|  | *GFRA2* | 1.0000 | 0.0000 |
|  | *AQP10* | 1.0000 | 0.0000 |
|  | *HHEX* | 0.9429 | 0.0048 |
|  | *NCS1* | 0.9429 | 0.0048 |
|  | *CHMP4B* | 0.9429 | 0.0048 |
|  | *FBXO3* | 0.9429 | 0.0048 |
|  | *CHAC1* | 0.9429 | 0.0048 |
|  | *GRHL2* | 0.9429 | 0.0048 |
|  | *EMC2* | 0.9429 | 0.0048 |
|  | *NPC2* | 0.9429 | 0.0048 |

**Table S12.** The mRNAs in each of the three networks, as well as the intersection and unique mRNAs across all networks.

| Networks | mRNAs |
| --- | --- |
| CeRNA network | *PLPP1, MT1G, DACT2, CHGA, CAMKK2, HMGN3, ARL4A, TRAM2, SRSF1, EREG, FRMD4B, SAR1B, BMF, NCS1, CHMP4B, FBXO3, KLF5, RBBP6, TAB1, RNF222, GALNT5, ARFGAP2, CHAC1, EIF3H, LOX, KIAA0232, EMC2, PSMA4, KLF2, SH3RF3, DLL4* |
| Cytoplasm-localized DElncRNA-SRSF1 interaction network | *PLPP1, FLT4, GRAP, DACT2, PI3, TOR1AIP2, CAMKK2, LRFN3, RPL37A, ZBTB2, SAMD4B, GSDMA, RNASEH2B, TRAM2, FRMD4B, AQP10, HBS1L, KLF5, RBBP6, TAB1, GALNT5, ARFGAP2, AMY2A, CHAC1, EIF3H, LOX, ST7, DLL4, BAIAP2L2* |
| Nucleus-localized DElncRNAs trans-regulation network | *PLPP1, TRAM2, RBBP6, CAMKK2, TAB1, SPRR2G, DLL4, TOR1AIP2, FRMD4B, ZBTB2, LYZ, ST7, BMF, NPC2, RPL37A, HBS1L, EIF3H* |
| Intersection mRNAs of three networks | *DLL4, EIF3H, TRAM2, FRMD4B, PLPP1, TAB1, CAMKK2, RBBP6* |
| Unique mRNAs of three networks | *TRAM2, RBBP6, CAMKK2, TAB1, SPRR2G, DLL4, TOR1AIP2, FRMD4B, ZBTB2, LYZ, ST7, BMF, NPC2, RPL37A, HBS1L, EIF3H, PLPP1, MT1G, DACT2, CHGA, HMGN3, ARL4A, SRSF1, EREG, SAR1B, NCS1, CHMP4B, FBXO3, KLF5, RNF222, GALNT5, ARFGAP2, CHAC1, LOX, KIAA0232, EMC2, PSMA4, KLF2, SH3RF3, FLT4, GRAP, PI3, LRFN3, SAMD4B, GSDMA, RNASEH2B, AQP10, AMY2A, BAIAP2L2* |
